# The Multifaceted Nature of GLP-1: Molecular Mechanisms and Signaling Pathways in Metabolic and Neurodegenerative Diseases

**DOI:** 10.3390/ijms27041886

**Published:** 2026-02-15

**Authors:** Małgorzata Katarzyna Kowalska, Ahmed El-Mallul, Weronika Hudecka, Joanna Elżbieta Lubojańska, Piotr Jan Lubojański, Sara Małgorzata Orłowska, Łukasz Bednarczyk

**Affiliations:** 1 Department of Applied Chemistry, Casimir Pulaski Radom University, Street Chrobrego 27, 26-600 Radom, Poland; 2 Department of Health Sciences and Physical Culture, Casimir Pulaski Radom University, Street Chrobrego 27, 26-600 Radom, Poland; a.el-mallul@urad.edu.pl (A.E.-M.); weronika.hudecka@gmail.com (W.H.); asia.majewska13@gmail.com (J.E.L.); sara.orlowska17@gmail.com (S.M.O.); bednarczyk.lukasz21@gmail.com (Ł.B.); 3 Specialist Hospital Named Dr. Tytus Chalubinski, 1 Adolfa Tochtermana Street, 26-600 Radom, Poland; piotrlubojanski@gmail.com

**Keywords:** GLP-1, GLP-1 receptor agonists, obesity, type 2 diabetes mellitus, cardiometabolic risk, appetite regulation, mesolimbic reward pathway, molecular pathways

## Abstract

The aim of this article is to present the current state of knowledge regarding the use of GLP-1 agonists in the treatment of type 2 diabetes, obesity, and other potential clinical indications, including neurodegenerative conditions. The article describes the characteristics of the diseases discussed, with particular emphasis on the pathophysiological mechanisms and the impact of metabolic disorders on the course of the diseases. In addition, the specific role of GLP-1 receptor agonists and their mechanisms of action leading to improved clinical outcomes were discussed, including their impact on molecular pathways involved in glucose metabolism regulation, inflammatory processes, carcinogenesis, and neuroprotection. Based on meta-analyses of available clinical trials, the evidence supporting the effectiveness of GLP-1 agonist therapy in glycemic control, weight loss, and improvement of metabolic parameters was synthesized. Additionally, potential benefits beyond the metabolic system are discussed, including neuroprotective effects and impact on patients’ cardiovascular profiles, as well as risks and adverse effects associated with the use of GLP-1 agonists. The collected data indicate the growing role of GLP-1 agonists as an innovative and effective therapeutic strategy, while emphasizing the need for further research in the context of new clinical indications.

## 1. Introduction

In recent decades, the incretin hormone GLP-1 (“glucagon-like peptide-1”) has gained the status of a key factor regulating carbohydrate homeostasis, appetite, and metabolic processes, with effects that significantly exceed its traditionally attributed incretin function [1,2]. Consequently, it has become the starting point for modern therapies for diabetes, obesity, and cardiometabolic diseases [2,3]. The concept that the human upper gastrointestinal tract secretes substances that influence pancreatic function and insulin secretion emerged at the beginning of the 20th century [4]. This was inspired by the discoveries of the first gastrointestinal hormones: secretin in 1902 and gastrin in 1905. In 1906, the hypothesis was formulated that the duodenal mucosa could secrete a substance that stimulates the pancreas, and attempts to treat diabetes with intestinal extracts, although unsuccessful, initiated further research in this direction [4]. Several years later, Zunz and LaBarre demonstrated in studies on dogs that intestinal extracts induce a decrease in glycemia resulting from the stimulation of the endocrine function of the pancreas, leading to increased insulin secretion—a phenomenon termed incretin action [4]. A breakthrough in the development of this concept came after the discovery of insulin in 1921 and the development by Yallow and Berson in the 1950s of a method for determining its concentration in plasma [4]. Further research focused on identifying the intestinal hormones responsible for this mechanism [4]. Among them, GLP-1, a product of the expression of the proglucagon gene (GCG) [5], gained particular importance. Initially, GLP-1 was described as an incretin hormone that increases insulin secretion in response to a meal. Over the years, however, it has been shown that its action extends far beyond the pancreas to encompass, among others, the nervous, digestive, cardiovascular, renal, and immune systems [5].

Glucagon-like peptide-1 is a peptide hormone composed of 30–31 amino acids, secreted primarily by L cells of the small intestine and some neurons of the central nervous system in response to food intake [3]. It is produced by post-translational processing of proglucagon by the enzyme prohormone convertase 1/3 (PC 1/3), which leads to the formation of biologically active forms: GLP-1 (7–37) and its amidated form, GLP-1 (7–36), which is predominant in humans [3].

GLP-1 exhibits a multifaceted spectrum of action with significant physiological and clinical significance. GLP-1 receptors (GLP-1R), encoded by the *GLP1R* gene on chromosome 6, belong to the family of G protein-coupled receptors (GPCRs) [3]. Their activation leads to an increase in cAMP (cyclic adenosine monophosphate) levels, which initiates a cascade of metabolic reactions. GLP-1Rs are found in the pancreas, gastrointestinal tract, heart, lungs, kidneys, brain, adipose tissue, and thyroid C cells, which explains the broad spectrum of action of this hormone [3]. In the context of its short half-life of approximately 2 min due to rapid degradation by the enzyme dipeptidyl peptidase-4 (DPP-4), a number of long-acting GLP-1 analogs have been developed [3,6]. The development of these molecules has opened up new therapeutic possibilities that are currently being intensively investigated [6].

This article provides a detailed analysis of the latest developments in the clinical use of GLP-1 analogs. It covers both novel forms of administration and therapeutic combinations, as well as expanding areas of research, including diabetology, cardiology, neurology, oncology, and psychiatry. The aim of this paper is to present GLP-1 as a modern therapeutic tool whose range of applications in various fields of medicine is steadily expanding, thus paving the way for innovative therapeutic strategies in many fields of medicine. The role of GLP-1 in regulating intracellular processes was also analyzed, with particular emphasis on its impact on molecular pathways responsible for controlling energy metabolism, modulating the pro-inflammatory environment, reducing oxidative damage, and supporting neuroprotective functions.

## 2. Mechanism of Action and Pharmacokinetic Profile of GLP-1 Receptor Agonists: From Molecular Signaling to Clinical Effects

GLP-1 belongs to a group of incretin hormones that are released from intestinal cells in response to food intake [7,8]. This phenomenon is known as the incretin effect, which means that after oral administration of glucose, the response of pancreatic beta cells is much stronger than after intravenous infusion of glucose at a similar blood glucose concentration [9]. This is due to two main incretin hormones: glucose-dependent insulinotropic polypeptide (GIP), secreted by cells in the duodenal mucosal, and glucagon-like peptide-1 (GLP-1), released from L-type enteroendocrine cells located in the distal small intestine and colon [6,9].

GLP-1 is a peptide composed of 30–31 amino acids. The enzyme dipeptidyl peptidase-4 (DPP-4) hydrolyzes the peptide bond between the alanine residue in position 2 and the next amino acid, which limits the half-life of GLP-1 to only about 2 min and leads to the formation of inactive forms of the peptide [6]. Consequently, only 10–15% of secreted GLP-1 reaches the systemic circulation [6].

GLP-1 secretion is biphasic and occurs mainly from L-type enteroendocrine cells located in the distal small intestine (ileum) and colon. The early phase occurs 15–30 min after a meal. The late phase occurs after 90–120 min. This mechanism is associated with the proximal-distal connection, which is regulated by neurotransmitters such as acetylcholine and neuropeptides (gastrin-releasing peptide) [6]. The same authors have shown that γ-aminobutyric acid (GABA) and glycine stimulate GLP-1 secretion, while somatostatin inhibits its secretion, and its blockade causes an eightfold increase in GLP-1 secretion.

GLP-1 stimulates insulin secretion after oral glucose loading, a process that is disrupted in patients with type II diabetes. The concentration of bioactive GLP-1 in fasting plasma is 5–15 pM, and after a meal it can increase to values up to three times, and sometimes five times higher [10]. The concentration of both hormones (GIP and GLP-1) increases approximately 15 min after a meal, reaches a maximum after 30–45 min, and returns to baseline values after 2–3 h [9]. The GLP-1 receptor (GLP-1R) is a seven-transmembrane G protein-coupled receptor (GPCR) expressed in various cells, including pancreatic β cells, the gastrointestinal tract, heart, skeletal muscle, liver, central nervous system, and immune system cells [11]. Upon binding to GLP-1, GLP-1R primarily couples to the Gs protein, leading to the activation of adenylate cyclase (AC) and an increase in cyclic adenosine 3′,5′-monophosphate (cAMP) concentration in the cell. Elevated cAMP levels activate protein kinase A (PKA) and guanine nucleotide exchange protein 2 (Epac2), which together mediate many further signaling processes [6,12].

In pancreatic β cells, PKA and Epac2 signaling leads to the closure of ATP-sensitive potassium channels, composed of Kir6.2 subunits forming the pore and SUR1 regulatory subunits. The closure of these channels depolarizes the cell membrane, resulting in the opening of voltage-gated calcium channels (VGCC), in particular L-type channels such as Ca_V_1.2 and Ca_V_1.3. The influx of Ca^2+^ also triggers calcium-induced calcium release (CICR) from the sarcoplasmic reticulum via IP_3_ receptors and ryanodine receptors (RyR), which further increases the concentration of Ca^2+^ in the cytoplasm. An increase in intracellular Ca^2+^ concentration, together with the action of PKA and Epac2 pathways, promotes insulin granule exocytosis via Ca^2+^-dependent SNARE protein complexes. The increase intracellular Ca^2+^ concentration, combined with the action of PKA and Epac2 pathways, promotes insulin granule exocytosis via Ca^2+^-dependent SNARE (soluble N-ethylmaleimide-sensitive factor attachment protein receptor) protein complexes. In this process, SNAP-25, Snapin, Rim2, and other components of the SNARE complex play a key role in preparing and connecting insulin granules to the cell membrane, enabling effective insulin secretion in response to GLP-1 [12,13,14,15].

In addition, other signaling pathways, such as CREB (cAMP response element-binding protein) and HIF-1α, are involved in GLP-1 receptor (GLP-1R) signaling, contributing to β-cell survival and proliferation and to increased glucose-dependent insulin secretion. CREB plays a particularly important role in mediating the long-term effects of GLP-1, as GLP-1-induced increases in cAMP not only stimulate insulin granule exocytosis via the Epac2/Rap1 pathway, but also increase insulin secretion via CREB-dependent transcriptional mechanisms. Furthermore, CREB is essential for the cytoprotective action of GLP-1 against cytokine-induced apoptosis, partly through the regulation of genes such as Cdkn1a and CITED2, suggesting the existence of a CREB–CITED2–p21 axis. Another important regulator of GLP-1 activity in β cells is HNF1α, a transcription factor essential for the proper expression of genes specific to β cells, including components of the SNARE complex and enzymes involved in insulin biosynthesis. HNF1α interacts with CREB and other cAMP-dependent factors to ensure the proper expression of genes critical for effective insulin release in response to GLP-1 [12,13,14,15].

**Figure 1:**
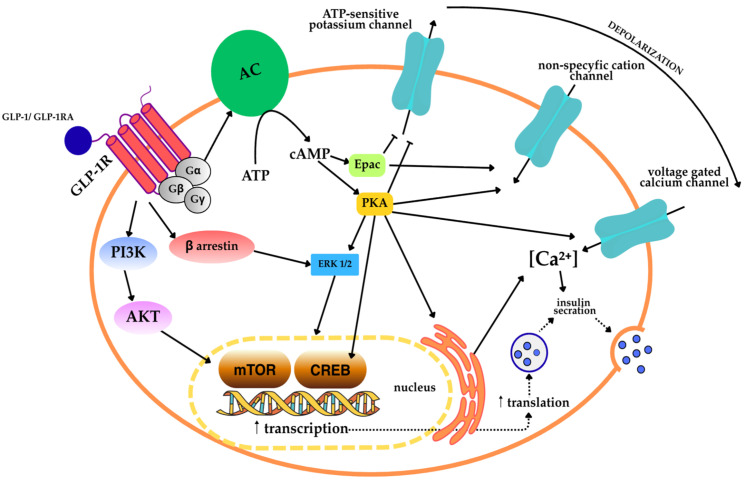
Mechanism of GLP-1-induced insulin secretion in pancreatic β-cells based on [12,13,14,16,17,18,19,20].

In summary, GLP-1R activation integrates multiple signaling mechanisms, leading to an increase in cytoplasmic Ca^2+^ concentration, promotion of insulin vesicle release, improvement of glycemic control, and protection of pancreatic β-cell function (Figure 1) [6,13].

Figure 1 shows the main intracellular signaling pathways activated by glucagon-like peptide-1 and GLP-1 receptor agonists in pancreatic β cells, which mediate glucose-dependent insulin secretion. The binding of GLP-1 to the GLP-1R, a seven-transmembrane receptor coupled to G protein, leads to the activation of stimulatory G protein, which results in the activation of adenylate cyclase (AC). The enzymatic activity of AC causes an increase in the intracellular concentration of cyclic adenosine 3′,5′-monophosphate (cAMP), which is formed from adenosine 5′-triphosphate (ATP). Elevated cAMP concentration activates two main signal effectors: protein kinase A (PKA) and guanine nucleotide exchange protein directly activated by cAMP (Epac). PKA phosphorylates numerous targets involved in the regulation of cell membrane excitability, including ATP-sensitive potassium channels. The activation of these channels leads to depolarization of the cell membrane, which promotes the opening of VGCC, allowing Ca^2+^ ions to flow into the cytoplasm. In parallel, PKA- and Epac-dependent signaling modulates non-selective cation channels, further enhancing depolarization and increasing Ca^2+^ influx. An increase in intracellular Ca^2+^ concentration initiates exocytosis of insulin-containing secretory granules via calcium-dependent SNARE protein complexes.

In addition to the acute effects associated with exocytosis, PKA-dependent signaling activates mitogen-activated protein kinase/extracellular signal-regulated kinase 1/2 (MAPK/ERK1/2) and phosphatidylinositol 3-kinase (PI3K)/protein kinase B (AKT) pathways, partly involving β-arrestin. AKT activation leads to stimulation of the mTOR (mammalian target of rapamycin) kinase pathway, which enhances protein synthesis and insulin biosynthesis. In addition, cAMP-dependent PKA activation leads to phosphorylation of the cAMP response element-binding protein (CREB) in the cell nucleus. CREB-dependent transcription increases the expression of genes involved in β-cell survival, insulin production, and secretory capacity, thereby supporting long-term insulin secretion and β-cell function.

In summary, signaling through GLP-1R integrates rapid, Ca^2+^-dependent mechanisms of insulin granule exocytosis with long-term transcriptional and translational responses mediated by mTOR and CREB, leading to enhanced glucose-dependent insulin secretion and preservation of pancreatic β-cell viability [12,13,14,18,19,20].

### 2.1. The Mechanism of Metabolic Signal Integration in the Brain and Appetite Control

**Figure 2:**
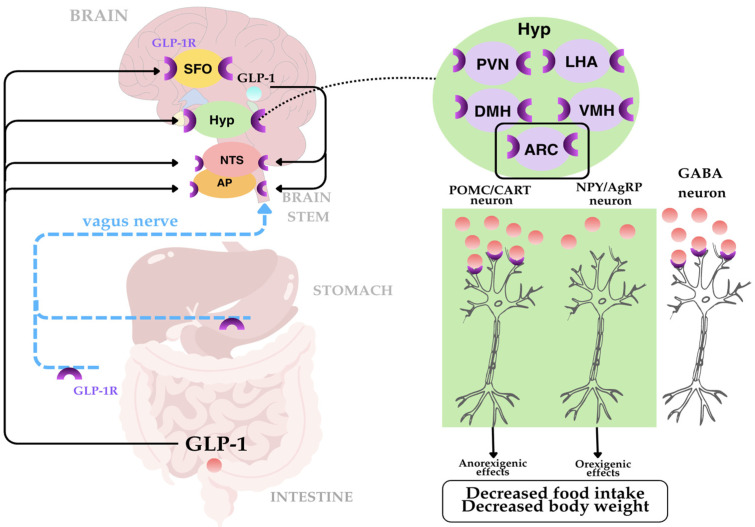
Hypothalamic and brainstem GLP-1 signaling in the control of feeding and energy balance based on [24,25].

GLP-1RAs bind to GLP-1 receptors, which are widely distributed in human tissues, including pancreatic islet cells, the heart, and the brain [8,10]. Food intake is regulated by numerous signaling pathways. The brain stem and hypothalamus, which receive and integrate peripheral signals, play an important role in this process [21]. In addition, the brain is a source of endogenous GLP-1, where it acts as a neurotransmitter. Numerous studies have analyzed changes in central nervous system activity in humans after administration of endogenous GLP-1 or GLP-1 receptor agonists, often in combination with food intake [22]. It is assumed that peripheral GLP-1 does not cross the blood–brain barrier [23]. The nucleus tractus solitarii, located in the brainstem, receives signals from the gastrointestinal tract regarding nutrient intake. GLP-1 receptor agonists bind to GLP-1 receptors in the solitary nucleus and increase the activity of serotonergic neurons, which increases the feeling of satiety [21]. Activation of GLP-1 receptors in the area postrema affects the dopaminergic system, weakening motivational stimuli associated with eating foods with high pleasure value, which leads to reduced appetite [21]. Another important structure to which signals are transmitted is the arcuate nucleus located in the hypothalamus. The arcuate nucleus contains anorexigenic (appetite-suppressing) and orexigenic (appetite-stimulating) neurons that regulate food control. Increased release of anorexigenic peptides and decreased release of orexigenic peptides in the form of neuropeptide Y lead to reduced food intake (Figure 2) [21].

Description of Figure 2: Glucagon-like peptide-1, which is secreted by intestinal L cells, exerts important central effects in the regulation of appetite and energy homeostasis through humoral and neuronal mechanisms. Peripheral GLP-1 signals are transmitted to the brain through the circulation and the vagus nerve, concentrating mainly in the nucleus of the solitary tract (NTS) and the area postrema (AP) of the brainstem. In addition, GLP-1 receptors are expressed in several brain regions involved in the control of metabolism, including the subthalamic nucleus (SFO), hypothalamus (Hyp), NTS, and AP. Within the hypothalamus, GLP-1R expression has been found in critical nuclei regulating feeding behavior, such as the paraventricular nucleus (PVN), lateral hypothalamic area (LHA), dorsal medial nucleus (DMH), ventromedial nucleus (VMH), and arcuate nucleus (ARC). In the ARC, GLP-1 directly activates anorexigenic proopiomelanocortin/cocaine and amphetamine-regulated transcript (POMC/CART) neurons and indirectly inhibits orexigenic neuropeptide Y/agouti-related peptide (NPY/AgRP) neurons through GABAergic neurons. Central GLP-1-dependent signaling integrates peripheral metabolic signals with hypothalamic and brainstem neural circuits, resulting in reduced food intake and body weight [24,25].

It has been noted that GLP-1 modulates the activity of serotonergic neurons in the mesolimbic system, including the ventral tegmental area (VTA) and the nucleus accumbens (NAcc), which may influence the regulation of reward-related and food-related behaviors. This results in an overall reduction in reward-based and hedonic eating behaviors [21]. Furthermore, there is evidence for the involvement of cytokines such as IL-6 and IL-1β in the transduction of GLP-1 anorexigenic signals in the hypothalamus and brainstem, highlighting the complex neurohormonal interaction in appetite regulation [22]. In studies in which GLP-1 agonist expression was selectively abolished in the dorsal-medial nuclei of the rat hypothalamus, reduced UCP1 (uncoupling protein 1) expression in brown adipose tissue, decreased brown adipose tissue temperature, reduced energy expenditure, and increased body weight and adipocytosis were observed [22].

Leptin, which is produced in adipose tissue and acts on the hypothalamus, plays an important role in satiety signaling, reducing appetite and increasing energy expenditure. GLP-1RAs enhance leptin signaling by reducing leptin resistance, which enhances the ability to suppress appetite [21].

GLP-1 and its agonists modulate a multi-level neural network in the brain, including the brain stem, hypothalamus, and reward system, affecting anorexigenic and orexigenic neurons, which leads to reduced appetite and food intake [8,10,21,23]. In addition, they increase the effectiveness of leptin signaling, enhancing appetite suppression and energy expenditure regulation, which together promote weight control and energy homeostasis [21].

### 2.2. Peripheral Mechanisms of Action of GLP-1 Agonists on Metabolism and the Cardiovascular System

**Figure 3:**
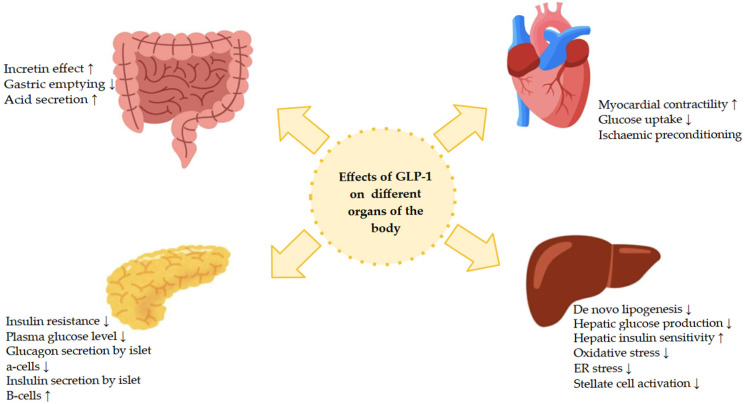
Effects of GLP-1 on different organs of the body based on [27,28]. ↑—increase/intensification of the secretion process. ↓—decrease of the secretion process.

Ghrelin, peptide YY, and cholecystokinin, as intestinal hormones, play an important role in regulating appetite and digestive processes. GLP-1RA modulates the secretion of intestinal hormones and enhances their peripheral action, which results in increased satiety. GLP-1 agonists reduce circulating ghrelin levels, which reduces appetite, food intake, and fat storage [21]. GLP-1 causes both short-term and long-term pleiotropic effects [23]. Within the pancreas, GLP-1 increases insulin gene expression and enhances glucose-dependent insulin secretion by inhibiting glucagon secretion in pancreatic alpha cells through the action of somatostatin [23,26]. Studies in mouse models of diabetes have shown that GLP-1 protects pancreatic β cells from glucolipotoxicity and diet-induced fatty liver disease, and improves hepatic glucose tolerance [23]. Furthermore, in vivo, GLP-1 increases glucose uptake, increases energy expenditure, and protects β cells [23]. Endogenous GLP-1 secreted by the intestine is absorbed into the liver via the portal vein. The liver is responsible for maintaining glucose homeostasis in the circulation through anabolic and catabolic processes of glucose and glycogen [26]. GLP-1 increases glucose uptake in the liver and reduces glucose-induced hyperglycemia, which remains dependent on the dose and rate of administration (Figure 3) [21,23].

Description of Figure 3: GLP-1 exerts diverse metabolic and extrapancreatic effects through activation of the GLP-1 receptor in multiple tissues. In the gastrointestinal tract, GLP-1 enhances the incretin effect, delays gastric emptying, and modulates gastric acid secretion. In the pancreas, it increases glucose-dependent insulin secretion by β cells, inhibits glucagon secretion by α cells, reduces insulin resistance, and lowers plasma glucose concentrations. In the liver, GLP-1 reduces de novo lipogenesis and hepatic glucose production, improves hepatic insulin sensitivity, and reduces oxidative stress and endoplasmic reticulum (ER) stress as well as the activation of hepatic stellate cells. In the cardiovascular system, GLP-1 affects myocardial contractility, glucose uptake, and ischemic conditioning. Together, these actions contribute to the overall metabolic and cardioprotective effects of GLP-1 [27,28].

The effects of incretins on insulin secretion and postprandial glucose regulation have been analyzed in particular [10]. Both GLP-1 and GIP bind to specific receptors on the surface of pancreatic β cells, stimulating glucose-dependent insulin secretion and inhibiting glucagon secretion. This effect disappears when glucose concentrations approach baseline values, which prevents hypoglycemia [8,9].

It has been shown that in healthy individuals, GLP-1 has an additive effect on glucose stimulation and increases the insulin response [10]. GLP-1 receptor agonists suppress glucagon secretion in a glucose-dependent manner and slow gastric emptying [7,21]. Slowing gastric emptying prolongs the process of digestion and glucose absorption, mitigating the postprandial increase in blood glucose and causing an earlier feeling of satiety, which contributes to a reduction in food intake [9]. In addition, they reduce appetite and increase the feeling of satiety due to their effect on the central nervous system [21]. This leads to a reduction in calorie intake and significant weight loss [21]. Their effect on the cardiovascular system has been proven, i.e., improvement of left ventricular ejection fraction, myocardial contractility, coronary flow, and reduction in the risk of cardiovascular events. Other proven effects of GLP-1 include increased glucose uptake in muscles and reduced glucose production in the liver [7]. Collins et al. indicated that GLP-1 analogs caused a reduction in overall mortality and a reduction in glycated hemoglobin by approximately 1%. The results of the above authors’ studies confirmed that GLP-1 receptor agonists reduce apoptosis of pancreatic beta cells and promote their proliferation [1].

### 2.3. Pharmacokinetic Characteristics, Forms of GLP-1 Receptor Agonists

**Figure 4:**
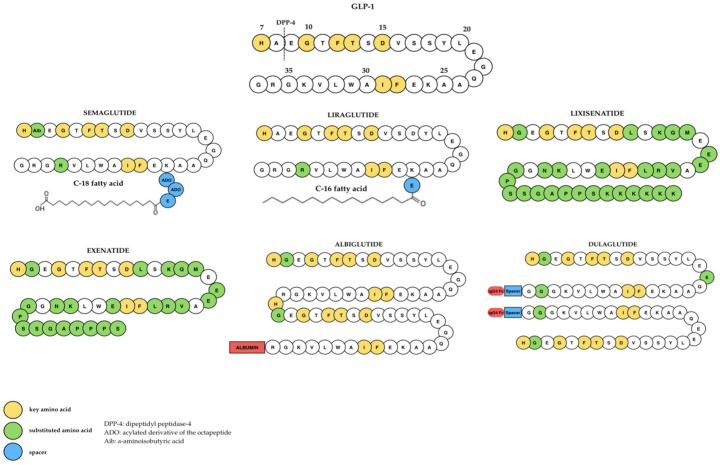
Structures and modifications of therapeutic GLP-1 analogs—peptide sequences of FDA-approved agonists based on [29,30].

GLP-1 receptor agonists are an important group of drugs recommended for the treatment of various diseases. GLP-1 receptor agonists (GLP-1RAs) are synthetic analogs of human GLP-1 with a more stable pharmacodynamic profile, allowing them to achieve greater effects than endogenous GLP-1 (Figure 4) [8].

This group has rapidly become the main class of drugs used in the treatment of type 2 diabetes and obesity [21]. GLP-1 receptor agonists are a new group of hypoglycemic drugs whose action is based on enhancing the incretin effect [9]. In 2010, the first two drugs in this group were registered for the treatment of type 2 diabetes: exenatide and liraglutide [9]. GLP-1 receptor agonists enhance insulin secretion in response to a carbohydrate-containing meal, which prevents postprandial hyperglycemia. At the same time, they reduce hepatic glucose production and slow gastric emptying. In addition, they affect the hypothalamic centers, reducing appetite and hepatic fat accumulation, which counteracts the development of fatty liver disease [9]. The action of GLP-1 receptors is strictly regulated. After ligand binding (GLP-1), homologous desensitization may occur, i.e., a temporary decrease in receptor reactivity through phosphorylation of serine and threonine residues in the C-terminal tail of the receptor, which limits cAMP production and activation of effector pathways. GLP-1 receptors may also undergo internalization, which involves their temporary removal from the cell membrane to endosomes, allowing for their subsequent resensitization or degradation. Under conditions of chronic metabolic stress, such as obesity or insulin resistance, downregulation of GLP-1 receptor expression in target cells is observed, which reduces sensitivity to GLP-1. On the other hand, in response to short-term exposure to GLP-1 or other stimuli, upregulation of receptors may occur, increasing their presence on the cell membrane and improving signaling efficiency. These regulatory mechanisms—desensitization, internalization/resensitization, and up- or down-regulation—form an integrated system of control of GLP-1R activity and influence the efficacy of GLP-1 agonist therapy in various physiological and pathological conditions [31,32]. Representatives of this group of drugs vary in terms of duration of action, chemical structure, pharmacokinetics, and physiological effects, which determines their therapeutic profile and dosage [6,8].

GLP-1RAs can be classified according to their pharmacological duration of action. Short-acting GLP-1RAs, such as exenatide and lixisenatide, cause short-term activation of the GLP-1 receptor (GLP-1R), while long-acting preparations, including liraglutide, semaglutide, and efpeglenatide, are characterized by a prolonged half-life, which allows for long-term activation of the GLP-1R [7]. Exenatide is a synthetic form of the naturally occurring peptide exendin-4, which was isolated from the saliva of the *Heloderma suspectum* lizard [6]. It shows 50% homology in its amino acid sequence with native GLP-1. It is resistant to the action of the enzyme dipeptidyl peptidase, and therefore does not undergo rapid inactivation [9]. The drug is administered subcutaneously 60 min before breakfast and dinner, and its main effect is to reduce postprandial glycemia [6]. After subcutaneous injection, its half-life is approximately 4 h, reaching peak concentration between 2 and 3 h, with the therapeutic effect lasting up to 7 h [9]. Exenatide is metabolized in the kidneys and liver through hydrolysis, which results in the formation of smaller and inactive peptides that are excreted in the urine [7]. After absorption, exenatide is rapidly eliminated by the kidneys after proteolytic degradation involving dipeptidyl peptidase-4 [6]. In order to ensure a longer therapeutic effect, a prolonged-release form of exenatide has been developed [6,7]. In this preparation, the active substance is enclosed in microspheres of a copolymer of lactic and glycolic acid, which causes slow and uniform release of the drug into the bloodstream [6]. Extended-release exenatide causes a significant reduction in fasting blood glucose levels after two weeks of therapy [6].

GLP-1 receptor agonists in the form of semaglutide or liraglutide are administered subcutaneously, which accelerates the absorption process, and the maximum concentration of these drugs is reached within a few hours [7]. After absorption, these drugs are characterized by a small volume of distribution, remaining mainly in the bloodstream. They have a particular affinity for pancreatic cells [7].

Liraglutide is an acylated derivative of human GLP-1. It is characterized by 97% homology with the native peptide [9]. Thanks to a structural modification, which involves the attachment of a C16 fatty acid residue (palmitic acid) to lysine at position 26, the molecule becomes resistant to degradation by dipeptidyl peptidase-4 and reversibly binds to plasma albumins. This allows the formation of a deposit at the injection site, from which the drug is released gradually, prolonging its duration of action [6,9]. Liraglutide is characterized by slow absorption from subcutaneous tissue over approximately 10–14 h and a long half-life of approximately 13 h, which allows the drug to be administered once daily [9]. Liraglutide is metabolized in various tissues of the body through proteolytic degradation processes, without the involvement of a single dominant metabolic pathway [6,7].

Semaglutide was developed based on liraglutide, but contains two amino acid modifications and a chemical modification at the lysine residue in position 26, which increases its stability and resistance to dipeptidyl peptidase-4 [6]. Semaglutide, which has a prolonged release (ER) profile, has a longer half-life compared to other preparations such as liraglutide or exenatide [7]. The half-life of semaglutide is approximately 47 h, which allows it to be administered once a week [6]. The drug is characterized by strong binding to albumin, which slows down its elimination [6]. As a polypeptide, semaglutide undergoes metabolic breakdown into amino acids with the participation of plasma and tissue proteases [7].

Efpeglenatide is a modern GLP-1 analog developed in South Korea using Long Acting Protein/Peptide Discovery Platform technology [6,33]. This technology allows the half-life of the drug to be extended from weeks to months. The coupling of the CA-exendin-4 peptide fragment with the Fc fragment of IgG4 immunoglobulin reduces the activity of the drug due to the action of anti-drug antibodies [33]. Due to the increased molecular weight of efpeglenatide, renal clearance is reduced and the duration of action is prolonged [6].

Lixisenatide is a 44-amino acid peptide amide at the C-terminus, with a structure similar to exendin-4 [6]. The main route of elimination of lixisenatide is through the kidneys, therefore this drug is contraindicated in patients with severe renal impairment [6].

Differences in the chemical structure and elimination mechanisms of individual GLP-1 receptor agonists determine their duration of action, bioavailability, and dosing frequency [6,7,9]. An overview of these differences is presented in Table 1. The authors mentioned above confirm that, thanks to structural modifications in the form of acylation or the use of polymer carriers, modern GLP-1 analogs enable a longer-lasting pharmacological effect and less frequent administration of the drug, which increases the effectiveness of treatment and patient cooperation.

## 3. Current Use of GLP-1 in Medicine and Treatment

The properties of incretins, such as glucagon-like peptide type 1 (GLP-1), have become the basis for the development of modern antihyperglycemic drugs, i.e., GLP-1 receptor agonists, also known as GLP-1 analogs [37]. The rapidly growing interest in this group of drugs stems from biochemical modifications that have significantly increased their potency, efficacy, and duration of activity compared to the natural form of GLP-1 [1,9,37]. Modern GLP-1 analogs are synthetic peptides with an extended half-life, achieved through changes in the amino acid sequence, making them resistant to degradation by the DPP-4 enzyme [37]. Their actions (anorexigenic and insulinotropic) include the previously mentioned stimulation of insulin secretion, inhibition of glucagon secretion, and reduction in hunger, which translates into effective glycemic control in the treatment of diabetes [34]. Additionally, GLP-1 receptor agonists contribute to weight loss and improve the lipid profile by increasing HDL cholesterol levels and lowering LDL and triglyceride levels [22]. This makes them useful not only in the treatment of type 2 diabetes, but also in the treatment of obesity and related diseases, such as cardiovascular diseases and nonalcoholic steatohepatitis (NASH) [22]. A meta-analysis of 10 randomized trials involving a total of 67,769 patients provided clear confirmation of the cardioprotective effect of GLP-1 receptor agonists, particularly in the type 2 diabetes population [38]. The analysis demonstrated that the use of GLP-1 analogs was associated with a significant 13% reduction in the risk of major adverse cardiac events and a 14% reduction in cardiovascular mortality [38].

### 3.1. Characteristics of Diabetes and Scientific Support for the Use of GLP-1 in Diabetes Treatment

Diabetes is a chronic metabolic disease characterized by hyperglycemia, i.e., elevated blood glucose levels [39]. Abnormal glucose levels can result from both impaired insulin secretion by the pancreas and a defect in the function of this hormone, when the body is unable to effectively use the produced insulin [39,40]. Lack of adequate glycemic control leads to numerous organ complications, including damage to blood vessels, nerves, the heart, kidneys, eyes, and feet, and can consequently be life-threatening [39,40]. Diabetes currently represents one of the greatest global health challenges of the 21st century [40]. According to data presented in the 11th edition of the Diabetes Atlas developed by the International Diabetes Federation, approximately 589 million adults aged 20–79 years live with diabetes, meaning that one in eleven adults is affected by this disease [40]. The prevalence of diabetes is increasing, with the greatest increase observed in low- and middle-income countries, where the disease is associated with risk factors such as unhealthy diet, overweight, physical inactivity, low education levels, limited access to healthcare, and social inequalities [39,40].

Over 90% of all diabetes cases are type 2 diabetes, the occurrence of which is strongly correlated with obesity, poor diet, low physical activity, and genetic predisposition [40]. According to the recommendations of the American Diabetes Association (ADA, 2025) and the Polish Diabetes Association (PDA, 2024), GLP-1 receptor agonists are particularly useful in this type of diabetes [41,42]. These drugs are recommended for adults with type 2 diabetes regardless of HbA1c levels, especially in cases of coexisting obesity and cardiovascular disease, including symptomatic heart failure with preserved ejection fraction (HFpEF), as well as in patients with high cardiovascular risk or diagnosed atherosclerosis [41,42]. Their use is also recommended in patients with chronic kidney disease, both with eGFR 20–60 mL/min/1.73 m^2^ and/or albuminuria, in order to improve glycemic control, slow the progression of CKD, and reduce cardiovascular risk, as well as in advanced stages of the disease (eGFR < 30 mL/min/1.73 m^2^), where GLP-1 RAs are the preferred therapeutic option due to their low risk of hypoglycemia and favorable cardiovascular profile [41]. In addition, their use should be considered in patients with type 2 diabetes and metabolic syndrome-associated steatohepatitis (MASLD), especially in those who are overweight or obese, as well as in patients with confirmed or high risk of metabolic-associated hepatitis (MASH), both as monotherapy and in combination with pioglitazone [41,43]. In adults without absolute insulin deficiency, GLP-1 receptor agonists are preferred over insulin therapy, while in patients requiring insulin, combination therapy with GLP-1 RA is recommended to improve glycemic control, limit weight gain, and reduce the risk of hypoglycemia [41].

Currently, six drugs belonging to the GLP-1 receptor agonists (GLP-1RA) are approved and widely used in the treatment of type 2 diabetes in Europe and worldwide [41,44,45]. According to data presented by the European Medicines Agency (EMA) and the American Diabetes Association (ADA), these include: dulaglutide, liraglutide, semaglutide, exenatide, lixisenatide, and tirzepatide, a multi-receptor agonist that, in addition to acting on GLP-1R receptors, also shows activity on GIP-R receptors [41,44,46,47]. All of the above-mentioned GLP-1 analogs are administered by subcutaneous injection, except semaglutide, which is the only one also available in oral form [48]. It is worth mentioning that albiglutide was also included in this group of known GLP-1 agonists, but on 29 October 2018, the European Commission withdrew its marketing authorization in the European Union for commercial reasons unrelated to safety [46]. Clinical trials of the seventh GLP-1 receptor agonist, taspoglutide, were discontinued in Phase III due to the high incidence of adverse events [45]. Similarly, trials of danugliprone, an oral, small-molecule GLP-1 receptor agonist developed by Pfizer Inc., were terminated [49]. In April 2025, the company announced the termination of its clinical development program after signals of an unfavorable safety profile emerged 4despite promising efficacy results in glycemic control and weight loss [49].

Other authors [9] indicate that the ideal antihyperglycemic drug used in patients with type 2 diabetes should be characterized by long-term efficacy, a low risk of hypoglycemia, and a beneficial effect on lipid metabolism and the cardiovascular system. Furthermore, this drug should promote weight loss while being safe and well-tolerated [9]. The complex, multisystemic nature of type 2 diabetes requires that the choice of therapy consider both glycemic control and broad metabolic and cardioprotective benefits [9]. In this context, the group of GLP-1 receptor agonists (GLP-1RAs) described in this article has gained particular importance. A network meta-analysis from 2023 [43] provides significant confirmation of their high efficacy and safety in the treatment of type 2 diabetes. The analysis included 76 randomized controlled trials involving 39,246 participants and 15 different GLP-1RA drugs, with an observation period of at least 12 weeks [43]. The study results clearly confirmed that all analyzed drugs demonstrated a statistically significant hypoglycemic effect compared to placebo. A significant reduction in both glycated hemoglobin (HbA1c) and fasting plasma glucose was observed, confirming the high efficacy of this group of drugs in improving glycemic control [43]. Among the evaluated drugs, tirzepatid proved to be the most effective drug in terms of glycemic control, resulting in the greatest reduction in HbA1c and fasting plasma glucose [43]. In turn, CagriSema, a combination of semaglutide and cagrilintide, demonstrated the strongest weight-reducing effect, resulting in an average weight loss of 14.03 kg, while tirzepatid ranked second, with an average weight loss of 8.47 kg [43]. Additionally, some GLP-1 agonists have been shown to have a beneficial effect on lipid profiles. In particular, semaglutide significantly reduced LDL and total cholesterol levels, confirming its effective action on lipid metabolism [43]. Despite its high therapeutic efficacy, the authors of the meta-analysis noted an increased incidence of gastrointestinal side effects, particularly with higher doses of GLP-1RAs. Nevertheless, the study results clearly confirm that GLP-1 receptor agonists are one of the most effective and comprehensive groups of drugs in the treatment of type 2 diabetes, combining a strong hypoglycemic effect with weight reduction and improvement of metabolic parameters [43].

In recent years, intensive research has been conducted on new drugs from the GLP-1 receptor agonists group, aimed at further increasing the effectiveness of therapy and improving the comfort of treatment in patients with type 2 diabetes. One of the most promising representatives of this group is orforgliprone, a small-molecule, non-peptide GLP-1 receptor agonist [50]. Unlike most currently used preparations requiring parenteral administration, orforgliprone retains high biological activity after oral administration [49]. A randomized phase 3 study demonstrated that the use of orforgliprone in adults with early-stage type 2 diabetes led to a significant reduction in glycated hemoglobin levels and body weight compared to placebo [50]. Furthermore, the drug had a favorable safety profile—no cases of severe hypoglycemia were reported, and adverse events were mainly mild gastrointestinal symptoms [50]. Another compound currently in clinical development is cotadutide, a multireceptor agonist (a compound that activates more than one type of receptor, producing synergistic biological effects) that simultaneously acts on the GLP-1 (GLP-1R) and glucagon (GCGR) receptors [51]. In clinical trials, cotadutide demonstrated multifaceted beneficial metabolic effects, including significant reductions in HbA1c and body weight, as well as improvements in liver transaminase parameters, type III collagen propeptide levels, and fibrosis-4 index. These results indicate the potential efficacy of cotadutide in the treatment of nonalcoholic fatty liver disease (NASH) [51]. Additionally, available data suggest that this drug may have a beneficial effect on renal function in patients with type 2 diabetes and chronic kidney disease, likely by improving metabolic and hemodynamic parameters [51].

### 3.2. Characteristics of Obesity and the Scientific Basis for the Use of GLP-1 Receptor Agonists in Its Treatment

**Figure 5:**
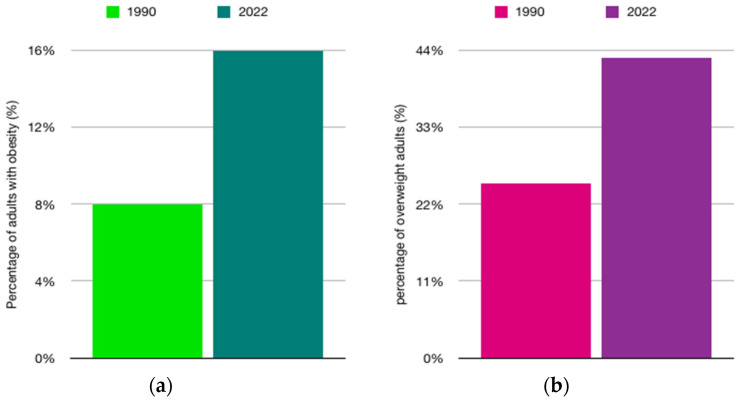
Prevalence of obesity (**a**) and overweight (**b**) in the adult population (1990–2022, WHO data) [52].

**Figure 6:**
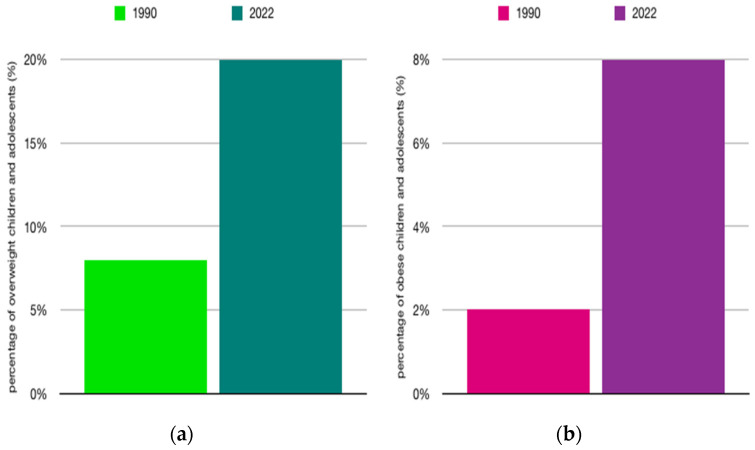
Prevalence of overweight (**a**) and obesity (**b**) among children and adolescents aged 5–19 years (1990–2022, WHO data) [52].

Obesity is among the most serious public health challenges facing our times. According to data from the World Health Organization (WHO), in 2022, one in eight people worldwide was struggling with obesity, and this rate has more than doubled among adults (aged ≥18 years) since 1990 (Figure 5a) and quadrupled among adolescents (Figure 6b) [52]. These data clearly highlight the scale and dynamics of the obesity epidemic, which has become a major health problem in the 21st century.

The basic tool used to assess and classify overweight and obesity is the body mass index (BMI) [53,54]. Its reference values vary by age and gender, particularly in infants, children, and adolescents [52,55]. BMI classification for adults and pediatric populations is presented in Table 2. Although BMI remains the most commonly used screening index, it should be remembered that it has significant limitations. The index does not reflect health status but rather measures the ratio of body weight to height [55]. Therefore, it should not be considered a diagnostic tool, but rather a guideline [55].

According to analyses presented in the World Obesity Atlas 2024, an elevated body mass index (BMI ≥ 25 kg/m^2^) is a significant risk factor in the context of the growing global health crisis [52,55]. It is estimated that of the approximately 41 million adult deaths annually caused by noncommunicable diseases, as many as 5 million cases are directly related to excess body weight [55]. The problem of excess body weight is also increasingly affecting the pediatric population. Current projections indicate that by 2035, over 750 million children and adolescents aged 5–19 years will be overweight or obese, which represents two in five children worldwide. Such a high BMI at a young age, as in adults, is associated with the risk of premature development of cardiovascular diseases, type 2 diabetes and strokes [55].

Obesity is a chronic, multifactorial disease characterized by excessive accumulation of adipose tissue, which leads to a number of serious health consequences that go far beyond esthetic issues [52,53]. It is considered one of the key factors in the development of numerous metabolic disorders and comorbidities, such as the previously mentioned type 2 diabetes, cardiovascular diseases, selected cancers, musculoskeletal disorders, and respiratory disorders [52,54]. It is worth emphasizing that pharmacotherapy for obesity is not recommended for all patients with an elevated BMI [56]. These medications should be considered in individuals who have not achieved sufficient weight loss despite lifestyle modifications, including changes in dietary habits, increased physical activity, and behavioral support [56,57]. According to recommendations, each medication used in obesity treatment should be combined with a reduced-calorie diet and regular physical activity, which increases the effectiveness of treatment and allows for maintaining the effects in the long term [57]. According to the National Institutes of Health (NIH) guidelines, pharmacotherapy can be implemented in adults if the BMI is ≥30 kg/m^2^, or ≥27 kg/m^2^ in the presence of comorbidities associated with overweight, such as hypertension or type 2 diabetes [56]. The exact BMI threshold values and inclusion criteria may vary depending on the type of medication used [57]. In the pediatric population, pharmacotherapy is not routinely recommended for children under 12 years of age [57]. Although available data indicate the possibility of obtaining beneficial therapeutic effects of GLP-1 analogs in this age group, the number of studies assessing their long-term efficacy and safety is still insufficient, which prevents the formulation of clear clinical recommendations [58]. The exceptions are clinical situations in which obesity is accompanied by serious comorbidities, for example in the case of orlistat, which can be used in children under 12 years of age with significant health problems, such as sleep apnea or orthopedic conditions [57].

According to the U.S. Food and Drug Administration (FDA), there are currently six drugs approved for long-term pharmacotherapy of overweight and obesity. These include phentermine–topiramate, orlistat, naltrexone–bupropion, tirzepatide, and the glucagon-like peptide type 1 receptor agonists (GLP-1 RA): liraglutide and semaglutide [47,56]. In the European Union, the European Medicines Agency (EMA) has approved all of these drugs for marketing, except the combination of phentermine and topiramate, which has not received EMA authorization. Four of these drugs: phentermine–topiramate, orlistat, liraglutide, and semaglutide, are approved for use in both adults and adolescents aged 12 years and older [56]. When using orlistat for the pediatric population, the recommended dose is 120 mg orally three times daily, taken with main meals containing fat or up to 1 h after a meal [59]. The most common side effects include gastrointestinal symptoms such as fatty stools, sudden urge to defecate, diarrhea, and abdominal pain, as well as impaired absorption of fat-soluble vitamins, which justifies supplementation with vitamins A, D, E, K, and β-carotene [59]. Liraglutide is approved for use in adolescents aged ≥12 years with obesity, defined as a BMI ≥ 95th percentile, with a body weight ≥ 60 kg. Treatment should be started at a dose of 0.6 mg daily, increasing gradually each week to a target dose of 3.0 mg daily [59]. In case of intolerance, it is possible to maintain a dose of 2.4 mg daily. An important limitation to the use of liraglutide is its contraindication in patients with medullary thyroid cancer or type 2 multiple endocrine neoplasia syndrome in their personal or family history [59]. Semaglutide is used in adolescents aged ≥12 years for the treatment of obesity, always in combination with dietary interventions and lifestyle modifications [60]. This drug is sometimes considered more effective than liraglutide in reducing BMI [60]. Treatment begins with a dose of 0.25 mg administered subcutaneously once a week, and the dose is then gradually increased depending on tolerance to 2.4 mg per week. The escalation schedule is as follows: 0.5 mg for 4 weeks, 1.0 mg for 4 weeks, 1.7 mg for 4 weeks, until the target dose is reached [60]. The combination of phentermine and topiramate can be used in adolescents aged ≥ 12 years with obesity (BMI > 95th percentile) in a gradual dose escalation regimen [59]. Treatment begins with the lowest dose (3.75/23 mg) for 2 weeks, then increases to 7.5/46 mg [59]. If weight loss is less than 3% after 12 weeks of therapy, further dose escalation to 11.25/69 mg for 2 weeks and then to a maximum dose of 15/92 mg is recommended [59]. Treatment should be discontinued if weight loss remains less than 5% after 12 weeks of maximum dose. In pediatric patients, therapy with this preparation may be associated with a risk of linear growth retardation, which requires regular monitoring [59]. Additionally, setmelanotide has been approved by the FDA for the treatment of adults and children aged 6 years and older, but only in patients with rare, genetically determined forms of obesity. This treatment is for patients with confirmed genetic mutations, including proopiomelanocortin (POMC) deficiency, proprotein convertase subtilisin/kexin type 1 (PCSK1), leptin receptor (LEPR), or Bardet–Biedl syndrome (BBS) [56]. Simultaneously, research is underway on new drugs with innovative mechanisms of action, primarily based on the incretin system, which may expand therapeutic options for obesity treatment in the future. FDA-approved drugs demonstrate varying efficacy and safety profiles, but numerous clinical trials have confirmed their beneficial effects on weight loss and improvement of metabolic parameters. Currently, GLP-1 receptor agonists and multi-receptor drugs, such as tirzepatide, are of particular interest, representing a modern approach to obesity treatment that goes beyond traditional strategies based on caloric restriction [47]. The mechanism of action of this group of drugs is related to the modulation of neuroendocrine processes involved in the regulation of appetite and metabolism. GLP-1R receptors, located in the hypothalamus, play a key role in the control of food intake by amplifying satiety signals, resulting in reduced appetite and weight loss [47].

Ongoing clinical trials are evaluating both the long-term effects and safety of already approved therapies and the potential of new incretin molecules under investigation [22,61,62,63]. The efficacy of liraglutide in the treatment of obesity has been confirmed in numerous clinical trials from the SCALE (Satiety and Clinical Adiposity—Liraglutide Evidence) program, including adults and adolescents [22]. Liraglutide treatment at a dose of 3 mg/day for 56 weeks in individuals with a BMI ≥ 27 kg/m^2^ (with comorbidities) or ≥30 kg/m^2^ led to a mean weight loss of 8.4 kg, with a reduction of >10% in over 33% of participants [22]. The treatment was associated with improvements in metabolic parameters, including HbA1c, blood pressure, lipid profile, and inflammatory markers [22]. Study discontinuation due to adverse events was reported in 9.9% of patients receiving liraglutide, compared with 3.8% of those receiving placebo. The most frequently reported adverse events were nausea, vomiting, and diarrhea [22]. Furthermore, the long-term results of the SCALE Obesity and Prediabetes study demonstrated that liraglutide reduces the risk of developing type 2 diabetes. After 160 weeks of the study, the disease was diagnosed in 2% of patients in the liraglutide group compared with 6% in the placebo group [22]. The efficacy and safety of liraglutide were also confirmed in an adolescent population with obesity [22]. In a 56-week study in patients aged 12–17 years, liraglutide was associated with significant weight loss (>5% in 43% of subjects), and the adverse event profile was consistent with previous observations in adults. These results confirm that liraglutide is an effective and well-tolerated treatment option for obesity in both adults and adolescents [22]. Semaglutide, a GLP-1 receptor agonist approved for the treatment of obesity and overweight, is administered weekly by subcutaneous injection (in the abdomen, thigh, or upper arm) [62]. Its clinical efficacy was demonstrated in a randomized, double-blind study of 1961 obese or overweight adults without diabetes [63]. Participants received semaglutide 2.4 mg weekly or placebo, in combination with a lifestyle intervention, for 68 weeks. The mean weight loss in the semaglutide group was 14.9%, compared with 2.4% in the placebo group [63]. A weight loss of at least 5% was achieved by 86% of individuals taking semaglutide and 32% of those in the control group, while a weight loss of ≥15% was achieved by 50% and 5% of the study participants, respectively. Treatment with semaglutide also led to significant improvements in cardiometabolic parameters and subjective assessment of physical fitness [63]. The obtained results confirm that semaglutide at a dose of 2.4 mg once weekly is a highly effective and well-tolerated GLP-1 receptor agonist, representing an effective therapeutic option in the treatment of obesity and overweight [63,64].

Currently, new generations of GLP-1 receptor agonists and their multi-receptor analogs, combining action on GLP-1, GIP, or glucagon receptors, are being intensively developed [65,66]. Many of them are in advanced phases of clinical trials [65], while some, including semaglutide, have already been approved for use in obesity treatment. Studies involving orforgliprone, retatrutide, efinopegdutide, and cotadutide, among others, are currently underway, and may expand therapeutic options in the future treatment of overweight and obesity [65,67,68]. Orforgliprone, discussed earlier in this article in the context of pharmacological treatment of type II diabetes, is the first oral, non-peptide GLP-1 receptor agonist currently in phase 3 clinical trials assessing its efficacy in obesity treatment [65]. A phase 2 study assessed its efficacy in obese or overweight adults without diabetes [64]. Participants took orforgliprone once daily at one of four doses (12, 24, 36, 45 mg) or placebo for 36 weeks [64]. After 26 weeks, the mean weight loss in the orforgliprone-treated groups ranged from –8.6% to –12.6%, compared to only –2.0% in the placebo group. After 36 weeks, the weight loss ranged from 9.4% to 14.7%, and 46–75% of orforgliprone users achieved at least a 10% weight loss, compared to only 9% of placebo participants [64]. The most common adverse events during this therapy were gastrointestinal in nature and mild to moderate, confirming the typical safety profile of GLP-1 agonists. The results of this study demonstrate that daily oral orforgliprone therapy effectively reduces body weight while maintaining a safety profile comparable to injectable GLP-1 agonists. Importantly, this drug has previously been described in the context of modern pharmacotherapy for type 2 diabetes, highlighting its potential as an innovative drug combining metabolic and weight loss benefits.

Another promising group of drugs that represent a new direction in incretin therapy are dual and triple receptor agonists, such as tirzepatide, retatrutide, efinopegdutide, and cotadutide [66]. These drugs affect different pathways regulating energy metabolism, resulting in greater weight loss and better control of metabolic parameters compared to classical GLP-1 agonists [66]. Retatrutide is a triple agonist of GLP-1, GIP, and glucagon receptors, which demonstrated significant weight loss in a phase 2 study in obese or overweight adults [67]. After 48 weeks of treatment, the highest doses of retatrutides (8 mg and 12 mg) led to a mean weight loss of 22.8–24.2%, respectively, compared with 2.1% in the placebo group [67]. The most common adverse events were gastrointestinal disturbances, which were dose-dependent and usually mild or moderate. These results indicate that retatrutide is a promising therapeutic option for the treatment of overweight and obesity, offering greater efficacy than traditional GLP-1 agonists [67]. Efinopegdutide is a single-molecule GLP-1 and glucagon agonist studied in individuals with obesity, type 2 diabetes, and MASLD/MASH. In phase 2 studies in obese nondiabetic individuals, 26 weeks of treatment with liraglutide at doses of 5–10 mg led to a mean weight loss of up to 11.8%, superior to liraglutide 3 mg (7.5%) and placebo (1.8%). The most common adverse events were gastrointestinal symptoms, with gradual dose titration significantly reducing treatment discontinuations due to adverse events. Efinopegdutide has been granted fast-track designation by the FDA for the treatment of MASH, highlighting its therapeutic potential as another GLP-1 agonist in the treatment of obesity. Efinopegdutide is a single-molecule agonist of GLP-1 and glucagon receptors, tested in patients with obesity, type 2 diabetes, and MASLD/MASH [68]. In phase 2 studies in obese nondiabetic individuals, 26 weeks of treatment with 5–10 mg doses resulted in mean weight loss of up to 11.8%, exceeding the results of liraglutide 3 mg (7.5%) and placebo (1.8%) [68]. Gastrointestinal adverse events were the most common, and gradual dose escalation reduced treatment discontinuations due to these events [68]. These results highlight the potential of efinopegdutide as another promising GLP-1 agonist therapy in the treatment of obesity. Survodutide, a GLP-1 and glucagon coagonist, is another multireceptor drug with potential in the treatment of obesity, currently in Phase 3 clinical trials [68]. In a Phase 2 study in obese individuals, it resulted in dose-dependent weight loss of up to 18.7% after 46 weeks, with gastrointestinal adverse events being the main reason for treatment discontinuation [68]. The drugs described above demonstrate modern pharmacotherapy options for obesity and overweight. It is worth noting that other promising drugs are also in clinical trials, such as mazdutide, pemvidutide, and cotadutide, which is discussed in this article in the context of the treatment of type 2 diabetes [68]

## 4. Potential New Therapeutic Pathways for GLP-1 Agonists—Current Research Directions

### 4.1. Cardioprotective Potential of GLP-1 Agonists

As mentioned earlier, glucagon-like peptide-1 (GLP-1) is an incretin hormone that plays a key role in regulating glucose homeostasis, but in recent years attention has also been drawn to its pleiotropic effects beyond the metabolic system. GLP-1 receptors (GLP-1R) are located not only in the pancreas, but also in the heart muscle, vascular endothelium, and nervous system, suggesting their significant involvement in the regulation of cardiovascular function. Current data from the literature indicate that GLP-1 receptor agonists (GLP-1RAs) exert a cardioprotective effect by improving endothelial function, reducing oxidative stress, inhibiting myocardial cell apoptosis, and exerting a beneficial effect on the lipid profile and blood pressure [69]. These effects translate into a reduced risk of cardiovascular events [69]. Among patients over 65 years of age with type 2 diabetes, it is estimated that as many as 68% of patients die from cardiovascular disease [69]. Chronic hyperglycemia in untreated or poorly controlled diabetes leads to endothelial dysfunction, increased oxidative stress, and activation of inflammatory processes that accelerate the development of atherosclerosis and vascular damage [70]. As a result, patients with type 2 diabetes are at a significantly higher risk of heart attack, stroke, and heart failure, and cardiovascular complications remain the leading cause of death despite advances in hypoglycemic therapy [69]. Effective cardioprotective effects have been demonstrated for individual GLP-1 RAs, including liraglutide, semaglutide, and dulaglutide [71,72,73]. The results of the study showed that semaglutide significantly reduces the risk of major cardiovascular events in patients with type 2 diabetes and high cardiovascular risk, reducing the combined rate of cardiovascular death, stroke, and myocardial infarction by 26% compared to the placebo group [73]. Similar effects were also observed with dulaglutide, the addition of which to the treatment of patients with type 2 diabetes for a period of 5 years also led to a reduction in the composite cardiovascular outcome, while lowering glucose levels, blood pressure, body weight, and the frequency of hypoglycemic episodes [72]. These results confirm that GLP-1 receptor agonists, i.e., semaglutide and dulaglutide, have multidirectional cardioprotective effects, independent of their glycemic effects [72,73].

The coexistence of diabetes and hypertension is an important pathogenetic factor in the development of diabetic nephropathy [74]. The combination of these two diseases significantly accelerates renal vascular damage, leads to progressive deterioration of renal filtration function, and increases the risk of end-stage renal failure. Diabetic kidney disease (DKD) is the most common cause of cardiovascular events in patients with type 2 diabetes, contributing significantly to increased mortality in this patient population [75]. Progressive kidney damage increases the risk of atherosclerosis, hypercoagulation, hyperlipidemia, and heart failure, highlighting the close relationship between kidney dysfunction and the pathogenesis of cardiovascular disease [75,76]. A meta-analysis conducted in 2022 by Mali et al. [77] showed that GLP-1RAs reduce proteinuria and improve overall kidney function. A potential mechanism for improving renal parameters is the induction by GLP-1 receptor agonists of phosphorylation and increased activation of the Na^+^/H^+^ exchanger type 3, which promotes the reabsorption of filtered sodium and thus improves renal hemodynamics. In addition, liraglutide has been shown to exert a nephroprotective effect by limiting extracellular matrix (ECM) deposition in the glomeruli. The action of liraglutide is associated with the regulation of the Wnt/β-catenin signaling pathway, which is responsible for the excessive production of proteins such as fibronectin, collagen IV, and α-SMA by mesangial cells [77]. Studies demonstrating the effect of GLP-1RAs indicate that their effect is primarily due to their multifaceted impact on the body’s metabolic function, resulting in the inhibition of nephropathy progression [74]. In one study conducted by Shi et al. in 2023 [78] confirmed that the development of diabetic nephropathy may be caused by excessive activation of inflammatory vesicles (NLRP3) and the accompanying specific form of apoptosis known as cell burns. A study conducted on mice showed that liraglutide significantly reduces inflammation and damage to podocytes in the kidneys of mice with type 2 diabetes. Protection of podocytes against inflammation did not occur in the control group after treatment with insulin degludec. Thus, the authors demonstrated that the protective effect of liraglutide may result from the regulation of the NLRP3–ASC–caspase-1 axis, which activates inflammatory cytokines and leads to podocyte damage [78].

There are reports indicating that GLP-1RAs also have a cardioprotective effect in people without carbohydrate metabolism disorders [79,80,81]. GLP-1 analogs are becoming increasingly important in the medical community, not only as antidiabetic drugs, but also as potential agents for the prevention and treatment of cardiovascular disease in the general patient population [79,80,81]. One of the first large clinical trials to evaluate the effect of GLP-1 receptor agonists in patients without diabetes was the SELECT trial, conducted in 2023 [80]. It demonstrated that the use of semaglutide significantly reduces the risk of cardiovascular events in people with obesity and cardiovascular disease, regardless of the presence of carbohydrate metabolism disorders. The study involved 17,604 patients with a BMI greater than or equal to 27 with cardiovascular disease, of whom three-quarters had a history of myocardial infarction. The study group was treated with semaglutide, which was administered subcutaneously once a week at a dose of 2.4 mg. Results presented by the authors [80] showed that over the 33-month study period, semaglutide reduced the risk of death from stroke, heart attack, and other non-fatal cardiovascular events by 20% compared to the placebo group. The beneficial effect of semaglutide observed in the study results is due to the multidirectional action of GLP-1RA, including reduction in inflammation, lowering of blood pressure, improvement of the lipid profile, and reduction in C-reactive protein levels. In addition, the drug promoted improved endothelial function and stabilization of atherosclerotic plaques, which translated into a reduced risk of cardiovascular events [80]. The effectiveness of GLP-1 receptor agonists in the treatment of cardiovascular diseases is also confirmed by the results of a meta-analysis conducted by scientists [82], which emphasize the need for further clinical trials to more fully assess their efficacy, safety, and scope of cardioprotective action. The analysis showed that GLP-1 agonists had a similar effect in overweight or obese patients without diabetes compared to patients with diabetes in terms of the risk of adverse cardiovascular events, all-cause mortality, and cardiovascular mortality. The results of the study clearly indicate that the benefits of using GLP-1 receptor agonists extend beyond the treatment of patients with diabetes, suggesting that their wider use in the population with cardiovascular diseases is justified [82].

### 4.2. Immunomodulatory Properties of GLP-1 Agonists

Among the autoimmune diseases in which GLP-1 receptor agonists have been shown to have beneficial effects are inflammatory bowel diseases (IBD). This group includes ulcerative colitis and Crohn’s disease. These conditions are characterized by impaired intestinal epithelial barrier function and chronic inflammation resulting from an abnormal immune response [83,84]. The goal of using GLP-1RAs, including liraglutide, dulaglutide, and exendin-4, is to reduce inflammation. The anti-inflammatory mechanism of action is based on the reduction in pro-inflammatory cytokines such as IL-2, IL-17a, IL-6, IFNγ, and TNFα, and the regulation of PI3K/AKT, NFκB, and CREB/PKA-dependent pathways [83,84]. Binding of the GLP-1 agonist increases cAMP and PKA activity, which inhibits the transcription of proinflammatory cytokines, such as TNF-α and IL-6, dependent on the transcription factor NF-κB, which regulates many inflammatory genes. At the same time, the involvement of the PI3K/AKT pathway contributes to cell survival and further suppression of inflammatory responses in immune cells [85,86]. In addition, additional benefits supporting the use of GLP-1 agonists in IBD include improvement of the intestinal microflora, weight loss, reduction in oxidative stress, and restoration of normal intestinal barrier function [83,84]. A study conducted in 2023 by Wang et al. [87] investigated the effect of GLP-1 on mice in which colitis was induced by administration of sodium dextran sulfate (DSS). According to the results presented by the authors, GLP-1 was shown to reduce inflammation induced in the macrophage line and reduce the inflammatory response by inhibiting the phosphorylation of molecules in signaling pathways such as AKT/NF-κB and MAPK. Furthermore, the use of DSS to induce an inflammatory response leads to increased intestinal epithelial permeability. GLP-1 may contribute to maintaining the integrity of the intestinal barrier and counteract damage to it by stimulating the synthesis of tight junction proteins, such as occludin and ZO-1, which limit abnormal immune responses in the intestinal mucosa [87]. The authors indicated that activation of the GLP-1 receptor increases the diversity of the colonic microflora. An increase in the number of Lactobacillaceae and Bifidobacteriaceae was observed, with a simultaneous decrease in Ruminococcaceae and Bacteroides. The growth of the Bifidobacterium population promotes the inhibition of colonization by potential pathogens, reduces the expression of pro-inflammatory mediators, and modulates the immune response [87]. The effect of individual GLP-1 analogs on the composition of the large intestine microflora was also confirmed by Gofron et al. in a study conducted in 2025 [88]. Based on the collected data, it was found that liraglutide promotes the growth of Alistipes and Butyricimonas species from the Bacteroidota phylum, which are important for metabolic functions. The diverse effects on the composition of the microbiome after the use of exenatide and exendin-4 have been confirmed in preclinical and clinical studies. In animal models, an increase in the abundance of bacteria that promote improved metabolism was observed. In contrast, human studies have reported both an increase in the proportion of microorganisms with beneficial metabolic effects and those associated with increased inflammatory responses. In the dulaglutide supply study, an increase in the abundance of the Lactobacillus strain is significant. In contrast, treatment with semaglutide led to an increase in A. muciniphila, with a simultaneous decrease in microbiota diversity, confirming that the effect of semaglutide on the intestinal microflora is more diverse [88].

GLP-1 analogs may also have a beneficial therapeutic effect in autoimmune and inflammatory joint diseases [89]. Rheumatoid arthritis is characterized by excessive inflammation, and in addition, these patients may experience mitochondrial dysfunction, increased oxidative stress, and collagen degradation due to the release of matrix metalloproteinases [90]. In a study conducted in 2019 by Du et al. [90], one of the GLP-1 receptor agonists was used to demonstrate its anti-inflammatory effect by modulating the activity of molecular pathways: JNK, AP-1, and NF-κB. After the application of lixisenatide at doses of 10 and 20 nM in human fibroblast-like synoviocytes (FLS) isolated from synovial tissues, a significant decrease in the concentration of TNF-α, IL-6, and IL-8 was observed, the presence of which results in exacerbation of pain, swelling, and joint damage. In FLS, there was a reduction in oxidative stress, which manifested itself through a decrease in reactive oxygen species (ROS) and 4-hydroxynonal, a product of lipid oxidation. In addition, reduced degradation of type II collagen, which is the main component of articular cartilage, has been demonstrated. This effect is caused by a reduction in the expression of MMP-1, MMP-3, and MMP-13 at the mRNA and protein levels after the use of lixisenatide [91]. In inflammatory joint diseases such as osteoarthritis (OA), GLP-1 analogs have been shown to exert multidirectional protective effects. As Meurot et al. pointed out in 2024 [91], the use of these compounds in OA is associated with additional effects on joint homeostasis, including anti-inflammatory, antioxidant, and chondroprotective effects. Liraglutide, one of the GLP-1 analogs, exhibits anti-apoptotic effects on chondrocytes through activation of the PI3K/Akt signaling pathway. In addition, GLP-1 analogs have been shown to exert protective effects by limiting macrophage infiltration in the synovial membrane. This mechanism includes, among others, the ability of liraglutide to reduce oxidative stress in macrophages by inhibiting lipid accumulation resulting from lipoprotein oxidation [91]. The above-mentioned authors also described the beneficial effects of another GLP-1 analog, exendin-4, which stimulates the proliferation, differentiation, and mineralization of bone cells in patients with osteoarthritis (OA). This effect is associated with the activation of the MAPK pathway and β-catenin signaling, which promotes osteogenesis, tissue repair, and maintenance of bone and joint homeostasis. All these mechanisms of action of GLP-1 analogs are aimed at limiting the degradation of joint structures. Although most reports remain at the stage of experimental research and early clinical trials, the results obtained provide a promising basis for the potential use of GLP-1 agonists in the treatment of inflammatory and autoimmune diseases of the musculoskeletal system [91].

In recent years, reports have emerged [92,93] indicating that GLP-1 receptor agonist therapy may modulate disease activity in patients with psoriasis. Psoriasis is a relapsing autoimmune disease [93,94]. The coexistence of obesity or type 2 diabetes is sometimes associated with the severity of psoriasis symptoms [92]. In patients with psoriasis or psoriatic arthritis, the prevalence of obesity is estimated at approximately 27–40% of cases [92]. In 2025, Siebert et al. [92] demonstrated a pathogenic relationship between the occurrence of obesity in patients and the severity of psoriatic lesions. In patients with coexisting obesity or type 2 diabetes who were treated with liraglutide, an improvement in PASI (Psoriasis Area and Severity Index) scores was observed after 12 weeks of therapy, resulting from a reduction in the expression of IL-17, IL-23, and TNF-α in psoriatic skin [92]. PASI is a measurement scale used to assess the severity of skin lesions in patients with psoriasis and is used to evaluate the effectiveness of therapy [94]. Numerous pro-inflammatory cytokines, including IL-17, IL-23, IL-12, IL-36, IL-6, and TNF-α, are responsible for the development of inflammation in psoriasis. They initiate and sustain the immune response, leading to keratinocyte proliferation and the formation of characteristic skin lesions [94]. Further reports on the efficacy of GLP-1 agonists come from a study published in 2025 [93]. The authors of the study [93] investigated the effect of semaglutide on the course of psoriasis in patients with type 2 diabetes in a study involving 31 participants. The study showed that in obese patients with psoriasis and type 2 diabetes, treatment with semaglutide for 12 weeks led, as in the case of liraglutide, to a significant decrease in the PASI index (from 21 to 10). In addition, patients experienced an improvement in quality of life as measured by the DLQI scale. Semaglutide therapy also reduced pro-inflammatory cytokine concentrations, CRP, BMI, and LDL cholesterol levels [93]. The above data and observations by the authors [92,93] indicate a significant impact of weight reduction in the treatment of psoriasis and psoriatic arthritis. The coexistence of obesity in psoriasis can lead to exacerbation of disease symptoms and increased expression of pro-inflammatory cytokines. Siebert et al. [92] showed that a reduction in body mass index (BMI) over a 10-year observation period was associated with a significantly lower risk of developing psoriatic arthritis compared to individuals whose BMI remained unchanged throughout the study period. These results confirm observations that the use of GLP-1 agonists for weight reduction may indirectly modulate the course of psoriasis [92,93].

### 4.3. The Role of GLP-1 Agonists in Modulating Central Nervous System Function

Endogenous GLP-1 synthesis is also observed in the brain, especially in hypothalamic neurons located in the solitary tract nucleus, the intermediate reticular nucleus, the piriform cortex, and the olfactory bulb [95]. In addition, GLP-1 receptors are also found in many regions of the brain, including the hippocampus, cerebral cortex, and hypothalamus, areas that are particularly susceptible to dysfunction and destabilization in the course of Alzheimer’s disease and Parkinson’s disease. Furthermore, the presence of GLP-1R receptors has been demonstrated in neurons, especially in cell bodies, dendrites and presynaptic terminals. The presence of numerous GLP-1 receptors in brain tissue indicates the potential for the use of GLP-1 analogs in neuroprotective therapy [96,97,98,99]. Activation of the GLP-1R initiates an increase in cAMP concentration, which regulates the PI3K/AKT and PKA/MAPK signaling pathways, affecting mitochondrial function, glucose homeostasis, and neuronal apoptosis processes. This results in structural and functional changes within the brain [97]. In the PKA pathway, activation of GLP-1R coupled with stimulating protein G leads to an increase in cAMP levels, which stimulates PKA kinase. Activated PKA then phosphorylates the transcription factor CREB, which increases the expression of neuroprotective genes such as BDNF and Bcl-2. This pathway enhances neuronal survival, promotes synaptic plasticity, and increases the resistance of nerve cells to excitotoxic stress [99]. In the parallel PI3K/AKT pathway, also activated by GLP-1R, protein kinase B (AKT) is phosphorylated, which inhibits proapoptotic factors such as Bad and GSK-3β. Increased AKT activity may further stimulate the mTOR pathway, leading to improved protein synthesis, cell growth, and metabolism. The interaction of signaling pathways promotes increased neuron viability in the CNS and may alleviate neurodegenerative processes [99]. In addition, the presence of GLP-1R signaling regulates metabolic activity by activating AMPK, which influences mitochondrial biogenesis and supports the maintenance of normal cellular energy production by mitochondria. At the same time, AMPK activation increases microglial phagocytosis, improving the energy balance of neurons and reducing the accumulation of neurotoxic protein aggregates [99].

In the scientific literature of the early 21st century, there has been an increase in the number of publications suggesting the neuroprotective properties of GLP-1 agonists in certain neurological and neurodegenerative diseases, such as Alzheimer’s disease, Parkinson’s disease, Huntington’s disease, memory disorders, epilepsy, multiple sclerosis, and peripheral sensory neuropathy [96,97,100,101,102]. The neuroprotective properties of GLP-1 are comparable to those of insulin, which acts as a growth factor in the brain, supporting neuron repair, stimulating dendrite growth and synaptogenesis, and protecting nerve cells from oxidative stress [101]. Insulin signaling disorders in the central nervous system contribute to the impairment of, among other things, the PI3K/AKT signaling pathway, which promotes tau protein phosphorylation, which may correlate with neurodegenerative changes in the brain and cognitive decline, including in Alzheimer’s disease [103]. The above-mentioned neuroprotective effects were confirmed in a publication by Hölscher in 2012 [101], presenting promising results from studies conducted on mice with models of central nervous system diseases. In the same publication, the author suggests that GLP-1 crosses the blood–brain barrier, protects memory formation processes and motor activity. In addition, GLP-1 supports the integrity and functioning of synapses, enhances neurogenesis, reduces apoptosis, protects neurons from oxidative stress, and reduces chronic inflammatory responses [101].

Patients with Parkinson’s disease (PD) experience disorders resulting from abnormal insulin signaling within brain structures, which is one of the causes of the pathogenesis of this disease. The cause of the dysfunction may also be chronic inflammatory stress, elevated levels of which have been demonstrated in patients with PD [104]. In light of current scientific reports, it has been noted that the development of insulin resistance is associated with a decrease in the number of dopamine transporters on the surface of neurons in the striatum and reduced dopamine release, which is a result of decreased insulin levels. Dopamine transmission disorders are responsible for the direct cause of the development of Parkinson’s syndrome [105]. Exendin-4, liraglutide, and lixisenatide have been shown to be effective in reducing symptoms and slowing disease progression, leading to clinically significant improvement in patients with Parkinson’s disease [104]. Although numerous scientific studies suggest that GLP-1 agonists effectively modulate molecular processes and nerve cell functions, research on their use in patients is still mainly at the clinical trial stage. One of the phase II studies is being conducted by Meissner et al. in 2024 [106]. A group of 156 patients participated in the study. The study examined the effect of lixisenatide on the progression of Parkinson’s disease in patients who had developed the disease less than 3 years prior to the start of the study and who had not experienced motor complications. After 12 months, patients receiving lixisenatide showed significantly less deterioration in motor function compared to the group of patients who received a placebo. Clinical assessment of patients was based on the Movement Disorder Society-Unified Parkinson’s Disease Rating Scale (MDS-UPDRS), which assesses the motor disability of patients with PD. Other researchers conducted a study evaluating the efficacy of exenatide in patients with moderate Parkinson’s disease, indicating its potential therapeutic effect [107]. After 12 months of observation, it was found that patients treated with exenatide achieved a score 3.5 points higher on the MDS-UPDRS scale than those receiving a placebo, indicating a more favorable improvement in motor function. The results obtained allowed the conclusion to be drawn that exenatide may also have neuroprotective potential and slow the progression of the disease [107]. Although the study cited suggests that exenatide may alleviate symptoms and slow the progression of Parkinson’s disease, there are also data questioning these results [108]. Contradictory data indicate the need for further clinical trials involving larger patient groups to prove the efficacy and potential of GLP-1 agonists in PD therapy. A similar mechanism of disease pathogenesis resulting from insulin signaling disorders also occurs in Alzheimer’s disease (AD), a disease characterized by irreversible neurodegeneration of neurons resulting in cognitive impairment [109]. The changes present in the brain tissue of AD patients are characterized by the loss of nerve cells, the presence of numerous neurofibrillary tangles, dystrophic nerve projections, and amyloid β precursor protein deposits characteristic of this disease. In addition, there is increased activation of genes and signaling pathways involved in cell apoptosis, impaired energy metabolism, mitochondrial dysfunction, and elevated parameters resulting from oxidative stress. Nerve cell dysfunction and the above-mentioned disturbances in metabolic and signaling pathways lead to neuron death through the induction of membrane lipid peroxidation and impaired function of membrane enzymes and glucose and glutamate transporters [109]. The not fully understood mechanism of Alzheimer’s disease pathogenesis is a significant limitation in the selection of effective therapies capable of modifying its course and slowing the progression of neurodegenerative changes. Current treatments for AD patients focus primarily on modulating cholinergic and glutamatergic transmission, thereby limiting the symptoms of the disease in order to improve quality of life [110,111]. Researchers have become interested in the use of GLP-1 receptor agonists as drugs that can affect cognitive processes by influencing glucose metabolism in the brain. It has been shown that glucose metabolism disorders are one of the factors leading to cognitive decline and the development of Alzheimer’s disease, and their regulation may contribute to slowing the progression of memory loss in patients with AD. This thesis is supported by the observation that some antidiabetic drugs improve the prognosis in patients with AD through their beneficial effects on glucose metabolism and cerebral vascularization and by reducing tissue inflammation [110]. In 2024, Wang et al. [112] published the results of a study containing data from over one million patients diagnosed with type 2 diabetes. The study showed that the use of semaglutide is associated with a 40% reduction in the number of first diagnoses of Alzheimer’s disease compared to other GLP-1RA drugs such as albiglutide, dulaglutide, exenatide, liraglutide, and lixisenatide, and a 70% reduction compared to other antidiabetic drugs. This effect was observed regardless of gender, age, and co-occurrence of obesity in patients [112]. A growing body of evidence suggests the promising potential of GLP-1 agonists in the treatment of Alzheimer’s disease, although full confirmation of their efficacy, mechanism, and safety requires further clinical trials [113].

Among other neurological diseases in which the neuroprotective effect of GLP-1 agonists has been observed in recent years is multiple sclerosis [114]. Multiple sclerosis (MS) is a neurodegenerative disease that results in demyelination and degeneration of axons in the central nervous system, leading to motor, cognitive, or sensory dysfunction. The symptoms of the disease are varied, depending on the location of the demyelination of neurons and loss of axons [115]. MS is also an autoimmune disease resulting from the failure of T cell tolerance mechanisms. One hypothesis for the development of multiple sclerosis is that autoantibodies directed against myelin components damage the myelin sheaths, causing inflammation in the CNS. This leads to secondary damage to axons and impaired nerve impulse conduction [114]. The aim of using GLP-1 in MS therapy is to exploit its anti-inflammatory and neuroprotective potential to bring the patient into remission and reduce the risk of recurrent attacks resulting from acute inflammatory episodes in the nervous tissue [114]. Sadek et al. in 2023 [115] conducted a study on mouse models in which autoimmune encephalomyelitis (EAE) was induced [115]. The study analyzed the effect of semaglutide on the motor function of mice with EAE and on inflammatory parameters assessed in a histopathological examination. Semaglutide was shown to reduce demyelination, oxidative stress, and neuroinflammation induced by EAE, as well as to enhance neuronal remyelination. The mechanism of action of semaglutide was based on stimulation of the PI3K/Akt pathway, which resulted in inhibition of GSK-3β. Inhibition of GSK-3β resulted in alleviation of the symptoms of neurodegeneration and neuroinflammation in the brain [115]. In 2025, Kashmaila et al. [116] published preliminary results of an assessment of the progression of multiple sclerosis in patients treated with GLP-1 agonists. The study compared two cohorts: patients with multiple sclerosis receiving subcutaneous or oral GLP-1 agonists and patients not receiving this therapy. The analysis of the results was based on scales assessing neurological functions. The results showed significantly greater disease progression in the group of patients not treated with GLP-1 agonists compared to the treated group. Dysfunctions in untreated patients included brainstem and cerebellar dysfunction and organ dysfunction, including bowel and bladder dysfunction [116]. The potential efficacy of GLP-1 agonist therapy, as well as its favorable safety profile in patients with multiple sclerosis, indicate the promising potential of this therapy in slowing disease progression and improving neurological function [116,117].

A number of mechanisms of action of GLP-1 receptor agonists in the central nervous system have drawn the attention of clinicians to their potential impact in the field of psychiatry. A growing body of evidence suggests that modulation of dopaminergic, glutamatergic, and GABAergic pathways, effects on the reward system, and regulation of brain centers may provide a basis for the use of GLP-1 analogs in the treatment of mental disorders such as addiction and eating disorders [98,118,119]. Data from previous studies suggest that the mechanism underlying the reduction in the desire to consume alcohol and other addictive substances may be related to the effect of GLP-1 agonists on reward and reinforcement mechanisms. Modulation of reward circuits via the GLP-1 receptor involves the amygdala and medial prefrontal cortex [98,119]. Klausen et al. in 2022 [118] indicate, based on their observations, that GLP-1 affects the brain centers responsible for pleasure and addiction, which may contribute to reducing the impulses associated with seeking addictive substances. In patients who abuse nicotine, GLP-1 agonists may affect the feeling of satiety, which indirectly reduces the desire to reach for cigarettes. This effect may be the result of action on the habenula, a brain structure that plays a role in regulating behavior, emotions, feelings of discomfort, and motivation, which in turn inhibits addictive behaviors. The same authors also point to a mechanism that occurs in cocaine addiction, where the use of GLP-1 agonists can activate a negative feedback loop. Under stress, corticosterone levels increase, which reinforces mechanisms that promote addictive behaviors, including an increased desire to use intoxicating substances. There are also many mechanisms indicating that GLP-1 receptor agonists may be beneficial in the treatment of psychiatric patients [118]. The first indications of the potential efficacy of GLP-1 receptor agonists in the treatment of alcohol dependence came from numerous preclinical observations conducted on animal models. Further observations on the possible efficacy of GLP-1RA therapy emerged from observations of patients with type 2 diabetes [120]. These individuals spontaneously reported that the use of liraglutide was associated with a reduced desire to consume alcohol. Subsequent studies have shown that drugs in this group, semaglutide and dulaglutide, can reduce the negative effects of alcohol consumption, such as excessive agitation, and alleviate withdrawal symptoms [120]. In addition, patients using dulaglutide as part of obesity treatment showed a significant reduction in alcohol consumption [120]. A study conducted at an American clinic between 2022 and 2024 by Hendershot [121] examined the effect of semaglutide on reducing alcohol consumption and alcohol cravings in adults with alcohol use disorder (AUD). The effect of semaglutide on alcohol reduction was examined in patients who did not want to undergo treatment and did not show a willingness to reduce their alcohol consumption. The results suggest that semaglutide reduced the number of drinks consumed during a drinking episode and reduced weekly alcohol cravings. Furthermore, as the observation period progressed, a greater reduction in heavy drinking was observed in the semaglutide-treated group than in the placebo group [121]. In addition, it was observed among participants who smoked cigarettes that treatment with semaglutide was also associated with a significant reduction in the number of cigarettes smoked [121].

This study suggests that GLP-1 analogs could be an equally effective form of therapy for AUD in the future, on par with drugs such as disulfiram, naltrexone, and acamprosate. The use of pharmacotherapy in patients undergoing addiction treatment and seeking to reduce their alcohol consumption may yield better results than in individuals who are not motivated to reduce their drinking [121]. However, further clinical trials are needed to confirm the efficacy of GLP-1 analogs and enable their future approval for the treatment of individuals with AUD.

Among other psychiatric disorders in which the use of GLP-1RA may have a beneficial effect, eating disorders are also mentioned [122]. The greatest therapeutic potential is observed in binge eating disorder (BED), especially when it leads to obesity [122]. In addition, binge eating may also co-occur with bulimia. GLP-1 receptor agonists in the hypothalamus act on POMC/CART and NPY/AgRP neurons, enhancing satiety signals and suppressing appetite, with the simultaneous involvement of serotonin pathways. Additionally, they modulate the activity of the hippocampus and prefrontal cortex, which affects emotional control and reduces compulsive overeating behavior [122]. The highest expression of GLP-1 receptors in humans is observed in the frontal cortex, indicating that GLP-1 signaling may involve higher-order cortical mechanisms in the regulation of eating behavior. This means that, in addition to controlling hunger, its action includes executive functions such as impulse control, decision making, and reward evaluation [119]. Current data and evidence suggest that GLP-1 receptor agonists may be a promising alternative to pharmacotherapy in bulimia and other psychiatric disorders whose underlying causes include both appetite regulation disorders and behavioral aspects related to eating control [123]. In 2013 [124], Mietlicki-Basse et al. noted that another potential mechanism of reward system stimulation following GLP-1RA administration is the activation of receptors in the ventral tegmental area (VTA) of the brain. Its role is to regulate motivation, pleasure, and reward processes, e.g., in the process of eating. Activation of GLP-1 receptors within the VTA reduces the consumption of tasty, high-fat foods mainly by limiting meal size. The anorexigenic effect is partly mediated by presynaptic glutamatergic signaling via AMPA/kainate receptors, with a simultaneous increase in dopamine neuron activity and dopamine production in the VTA [124]. In 2023, Richards and colleagues [125] conducted a study confirming the observation that the use of GLP-1 agonists, including semaglutide, may in the future be part of the treatment of binge eating disorder. In a group of patients diagnosed with BED, the efficacy of semaglutide was compared with that of drugs commonly used to treat this disorder, such as lisdexamfetamine and topiramate [125]. In addition, the effects of combination therapy, in which semaglutide was administered together with one of the above-mentioned drugs, were evaluated [125]. Treatment with semaglutide resulted in a significantly greater reduction in scores on the Binge Eating Scale (BES) compared to lisdexamfetamine and topiramate, and the combination of semaglutide with other drugs did not provide additional benefits [125]. These results confirm earlier reports that GLP-1 RAs, by affecting central mesolimbic pathways, including the satiety and reward systems, can effectively reduce BED symptoms [125]. Obesity is also a disease resulting from psychiatric disorders, and it is estimated that up to 30% of patients with obesity have problems with binge eating disorders [126]. Robert et al. in 2015 [126] observed obese patients using liraglutide for 3 months. The subjects were divided into two groups, one of which used liraglutide, diet, and exercise for 12 weeks, while the control group only followed a diet and performed the recommended physical activity [126]. In the study, 81% of those receiving liraglutide experienced a reduction in overeating behaviors. In addition, patients in the study group showed a reduction in body weight, waist circumference, and other parameters associated with increased cardiovascular risk, which may be an indirect effect of reducing compulsive binge eating [126]. Compared to previously used methods, such as cognitive behavioral therapy or treatment with SSRIs, treatment with liraglutide has been shown to not only improve binge eating, but also result in significant weight loss and have a beneficial effect on metabolic parameters. This indicates the potential for higher efficacy of therapy in this group of patients [126].

### 4.4. GLP-1 Agonist in Oncology—Potential Risks and Therapeutic Prospects

From the perspective of oncology and the risk of developing malignant tumors, the results of studies on the effects of GLP-1 receptor agonists are inconclusive. The literature indicates a reduced risk of certain obesity-related cancers, such as colorectal cancer and endometrial cancer [127,128,129]. In contrast, in the case of kidney and thyroid cancer, there are inconclusive signals suggesting a possible increase in the risk of their occurrence [127,130]. The effects of GLP-1RAs may result from their indirect or direct action on molecular mechanisms, modulation of the immune system, metabolic regulation, or influence epigenetic changes [131]. The mechanisms responsible for the anticancer effect focus on inhibiting the proliferation of cancer cells, inducing their apoptosis, and inhibiting the migration and invasion of cancer cells [131]. Activation of GLP-1R receptors increases cAMP levels, which leads to the inhibition of cancer cell proliferation by blocking the ERK pathway and activating the PKA-AMPK cascade, which inhibits mTOR and stops the cell cycle. In addition, p38 activation and CREB phosphorylation promote apoptosis induction, which is one of the mechanisms responsible for the anticancer activity of GLP-1 agonists [131]. Another mechanism underlying the inhibition of carcinogenesis is the anti-inflammatory effect of GLP-1RAs [132]. They inhibit chronic inflammation, which is considered a key factor promoting mutations, proliferation, angiogenesis, and cancer metastasis. The reduction in inflammation results from the modulation of macrophage activity and the suppression of the NF-κB pathway responsible for the expression of numerous pro-inflammatory genes. In this way, GLP-1RA prevents the creation of an environment conducive to tumor development and progression [132]. A study published in 2025 by Dai et al. [127] included 86,632 adult patients with obesity, half of whom were treated with GLP-1RA, while the other half constituted a control group consisting of individuals who were eligible for the drug but did not take it. The authors of the study [105] sought to determine the incidence of 14 types of cancer in patients, including liver, thyroid, pancreatic, bladder, colon, kidney, breast, endometrial, meningioma, upper gastrointestinal tract, ovarian, multiple myeloma, prostate, and lung cancers. The study found that people using GLP-1 receptor agonists had an overall lower risk of cancer compared to those not using GLP-1RAs. The greatest reduction in the risk of carcinogenesis was observed for endometrial, ovarian, and meningioma cancers, while a slightly higher risk of kidney cancer was noted [127]. Similar observations were made in 2024 by Wang et al. [133] after evaluating a group of 1.6 million patients with type 2 diabetes over a period of 15 years. The study aimed to assess the association between GLP-1RA use and the risk of obesity-associated cancers (OAC) in patients with type 2 diabetes compared to insulin or metformin. The results also confirmed the protective effect of GLP-1 agonists in 10 of the 13 cancers studied: including colorectal, pancreatic, liver, gallbladder, meningioma, ovarian, esophageal, multiple myeloma, endometrial, and renal cancers compared to patients using insulin [133]. In addition, the study also observed an increased risk of kidney cancer in patients using GLP-1RA compared to those taking metformin [133]. Apart from a few studies, including the 2025 study by Dai et al. [127], which suggest a marginally increased risk of kidney cancer, there is no specific data confirming the mechanisms of cancer development in this organ. Furthermore, the study [133], which indicated a possible increase in the risk of kidney cancer after the use of GLP-1RAs, was based on an analysis of a dataset that lacked detailed clinical information, including drug exposure, confounding factors, and tumor type and stage, which limits the ability to draw reliable conclusions [133]. In a study published in 2025, Wang [134] investigated the effect of semaglutide on inhibiting the progression of squamous cell carcinoma of the oral cavity. The study showed that GLP-1R expression is significantly elevated in oral squamous cell carcinoma (OSCC) cells compared to normal oral keratinocyte (NOK) cells. The increase in GLP1 receptor expression in OSCC cells demonstrates the efficacy of semaglutide. Semaglutide was shown to inhibit OSCC cell proliferation, migration, and invasion and induce apoptosis, which was associated with the activation of the P38 MAPK signaling pathway. These results indicate that semaglutide may exert a direct antitumor effect in OSCC by modulating molecular pathways [134].

Another cohort study evaluating the effect of GLP-1 receptor agonists (GLP-1RAs) on carcinogenesis was published in 2024 by Wang et al. [128]. The study involved observing more than 1.2 million patients with type 2 diabetes for the occurrence of colorectal cancer who were treated with GLP-1RA. It is worth noting that the patients studied had not previously been treated with other antidiabetic drugs, which further increases the reliability of the results obtained regarding the impact and effectiveness of GLP-1RA. The analysis showed that the use of GLP-1 receptor agonists was associated with a significantly lower risk of developing colorectal cancer (CRC) compared to patients treated with insulin or other antidiabetic drugs [128].

A stronger preventive effect against CRC has been demonstrated in obese or overweight patients, indicating a possible mechanism related to both weight loss and other weight-independent effects [128]. Obesity in patients is often associated with adverse risk factors for cancer development, and therefore more and more studies are looking for mechanisms by which GLP-1 agonists can reduce the risk of cancer, both through indirect effects related to weight loss and mechanisms independent of it [127,128,133]. In obese patients, adipose tissue undergoes remodeling, accompanied by abnormal secretion of pro-inflammatory factors and adipokines that promote the proliferation or invasion of cancer cells in tissues [131]. At the same time, there is a decrease in adiponectin, whose reduced concentration has a carcinogenic effect. As a result of the changes occurring in obesity, increasing leptin levels result in increased systemic inflammation, inhibit apoptosis, and impair immune system function. These actions promote the formation of cancer cells [131]. Thyroid cancer is the most common cancer identified as a neoplastic disease and is the most common complication of GLP-1RA use. The literature includes both studies that show no association between GLP-1RA use and an increased risk of thyroid cancer and studies suggesting their carcinogenic potential [135,136]. In 2023 [130], Bezin et al. conducted an analysis of a French database of patients with type 2 diabetes to assess the association between the use of GLP-1 receptor agonists and the risk of developing thyroid cancer. The study included 2562 patients diagnosed with thyroid cancer, who were compared with 45,184 individuals in the control group. The results of this study led to the conclusion that the use of GLP-1RAs for 1–3 years was associated with an increased risk of thyroid cancer and medullary thyroid cancer [130]. The finding of an increased risk of thyroid cancer in GLP-1RA users may have been due to more frequent and intensive medical monitoring of these patients. As with the data indicating kidney cancer, the observational nature of the study, including the lack of complete control over confounding factors during GLP-1RA therapy, may have contributed to an overestimation of the actual number of thyroid cancer cases after GLP-1RA use. Observations regarding an increased risk of thyroid cancer were also published in 2024 by Silveria et al. [137]. Observations regarding the increased risk of thyroid cancer are also confirmed by a study published in 2024 by Silverii et al. [137]. The meta-analysis reviewed 64 studies, including 26 in which at least one case of thyroid cancer occurred. The authors confirmed that GLP-1RA treatment was associated with an increased risk of overall thyroid cancer [137]. In the meta-analysis by Silveria et al. [137], 38 of the included randomized trials did not report any cases of thyroid cancer in the analyzed patient groups, which is a common occurrence in the analysis of rare events and significantly limits the precision of reliable risk estimation. Such a large number of data with zero events makes the results susceptible to change with the appearance of even a few new cases in subsequent studies. Consequently, study results suggesting a possible increase in the risk of thyroid cancer should be questioned and confirmed in studies with longer follow-up times and a larger number of events [137]. Interest in the context of a potential increase in thyroid cancer risk has focused on demonstrating that thyroid C cells in neoplastic lesions express GLP-1 receptors. The use of GLP-1RA therapy led to the activation of these receptors, which initiated tissue hyperplasia and increased calcitonin synthesis [138]. This mechanism was initially interpreted as a potential pathway initiating carcinogenesis in thyroid cancer, but to date there is no conclusive scientific data confirming a causal relationship between the use of GLP-1RAs and the pathogenesis of thyroid cancer [138,139]. In an analysis conducted by Brito et al. in 2025 [136], the authors questioned the actual increase in the risk of thyroid cancer during GLP-1RA use. They pointed out that the observed increase in the detection of neoplastic changes in patients treated with GLP-1RAs may be due to greater vigilance on the part of clinicians and more frequent imaging tests in this group, which led to a higher detection rate compared to patients using other antidiabetic drugs. The authors noted a higher frequency of detection of lesions in the first year of therapy, confirming the possible impact of intensive monitoring of patients treated with GLP-1RAs. These results prove that the observed increase in thyroid cancer diagnoses in this group of patients may be due to more frequent diagnosis rather than the direct effect of GLP-1 agonists on the process of thyroid tissue carcinogenesis [139].

The results of studies to date, focusing primarily on the last decade, indicate that the role of GLP-1 receptor agonists in carcinogenesis is not clear [135,136]. Both their potential tumor-suppressing effect and possible carcinogenic effect require further verification in subsequent clinical trials.

**Figure 7:**
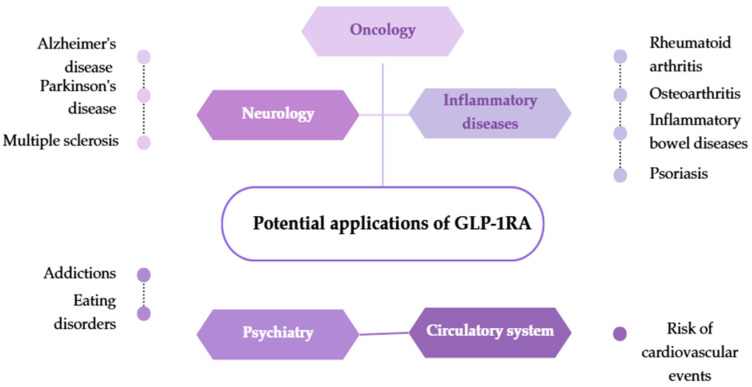
Potential applications of GLP-1RA agonists [71,72,73,83,84,90,93,101,106,109,112,115,120,122,123,127,128,133].

The data collected on the potential uses of GLP-1 receptor agonists (GLP-1RAs) indicate their versatile therapeutic potential. Unfortunately, apart from their use in the treatment of obesity and type 2 diabetes, where these drugs are already routinely used in clinical practice, other potential areas of use are based mainly on the results of clinical and preclinical studies. Despite numerous promising reports in the fields of neurology, autoimmune diseases, cardiology, and oncology, the role of GLP-1 agonists in these areas remains the subject of intensive research (Figure 7). This highlights the need for further multicenter clinical trials to fully determine the efficacy and, above all, the safety of GLP-1RAs in these disease entities, thereby establishing their place in future guidelines available to clinicians.

In order to facilitate and accelerate verification and to improve the accessibility of the information, a summary of the authors’ studies along with their key conclusions is provided in Table 3.

## 5. Safety Profile and Potential Adverse Effects of GLP-1 Agonist

In the clinical trials analyzed, a recurring trend can be observed in which patients discontinued participation in the trial due to adverse events. Data collected in a 2024 analysis by Sharma et al. [140] indicate that approximately 10% of patients discontinued semaglutide therapy, compared to 3% of patients in the placebo group [140]. Disorders such as vomiting, abdominal pain, diarrhea, and nausea were the most common group of complaints experienced by patients using GLP-1RAs [141]. Frequent adverse events make it difficult for clinicians to obtain complete and reliable clinical data from the study. In a meta-analysis by Xie et al. in 2025 [141] estimated that the total incidence of gastrointestinal adverse events affected as many as 11.66% of patients. Patients most often reported nausea (21%) and least often reported decreased appetite (approximately 5%). Tirzepatide, a simultaneous GLP-1 and GIP receptor agonist, was associated with the highest risk of adverse effects, including nausea and diarrhea. Dulaglutide and exenatide, on the other hand, were relatively better tolerated [141]. In addition to gastrointestinal side effects, the use of GLP-1RAs may be associated with pancreatitis, headaches, or allergic rashes, but their frequency and clinical significance are much lower compared to gastrointestinal effects [142]. The authors [143] demonstrated a correlation between the dose of GLP-1 used and the risk of adverse effects, which may have a significant impact on the high percentage of patients discontinuing treatment. In a study by Ma et al. in 2023 [143], it was shown that semaglutide at a dose of 2.4 mg was most effective in achieving at least 5% weight loss (OR 10.88), while the 1.0 mg dose differed slightly in effectiveness (OR 7.02). The use of semaglutide at a dose of 2.4 mg was associated with the highest risk of adverse events. Considering the safety profile, the use of a lower dose in the context of >5% weight loss would be comparably effective and associated with a lower risk of adverse events [143].

Due to the risk of adverse events, it is recommended that patients be monitored, especially in the initial period of treatment, during the critical period of treatment tolerance. An analysis conducted in 2024 by Sharma et al. [140] indicated that the incidence of serious adverse effects is estimated at 8.9% in patients treated with semaglutide and 6.2% in patients treated with liraglutide. Among the more significant adverse events are pancreatitis, gallstones, renal failure, and thyroid cell hyperplasia. A study conducted in 2025 by Tamilwanan et al. [144] showed that the incidence of serious adverse events in patients using semaglutide ranged from 7% to 13.4%. These results were comparable to those in the group of patients who did not receive GLP-1 analog treatment. Due to the comparable incidence of serious adverse events in the GLP-1 analog group and the placebo group, the study indicates a favorable safety profile for these drugs [140].

The authors [140,144] mention an increased incidence of cancer in the group of patients taking GLP-1 analogs compared to patients using placebo, but there is no specific data on the possible mechanism. GLP-1 cannot be clearly identified as a potential carcinogen. Furthermore, the data come from patients in whom GLP-1 was used for specific conditions, including obesity, which may also predispose to the development of cancer [140]. Currently, to ensure patient safety, the use of GLP-1 receptor agonists is contraindicated in individuals with a clinical history of medullary thyroid carcinoma (MTC) and in patients with multiple endocrine neoplasia type 2 (MEN2) [140].

In order to reliably demonstrate the long-term safety of GLP-1 agonists, further clinical studies involving patient observation are necessary. Currently available data mainly concern observation periods of several years, which are insufficient to fully assess the potential long-term effects of this class of drugs.

## 6. Conclusions

GLP-1 receptor agonists are currently an important component of obesity and type 2 diabetes therapy, with documented efficacy in improving glycemic control, reducing body weight, and lowering cardiovascular risk. A growing number of studies indicate their beneficial effect on metabolism in general, including improved insulin sensitivity and lipid profile, confirming their role in the comprehensive management of metabolic disorders. Clinical analyses also suggest the potential for using GLP-1 therapy in the treatment of patients with related pathologies, such as insulin resistance, hypertension, and dyslipidemia, and future research may extend its use to neurodegenerative and neurological diseases, including Alzheimer’s disease and epilepsy, where modulation of metabolic and neuroprotective pathways may bring significant therapeutic benefits. However, it should be noted that further research is needed to determine the optimal treatment regimens, long-term efficacy, and safety of GLP-1 therapy in both adult and pediatric populations, as well as in the context of a wide spectrum of metabolic and neurological pathologies. In light of the accumulated data, GLP-1 appears to be a promising tool not only in the treatment of obesity and diabetes, but also in future therapeutic strategies involving metabolic and neurodegenerative diseases.

The preparation of this type of publication serves as a comprehensive compendium of knowledge, supporting medical staff and patients in making informed therapeutic decisions.

## Figures and Tables

**Table 1:** Pharmacokinetic characteristics, formulations, dosing, clinical indications, and chemical structures of GLP-1 receptor agonists based on [6,10,12,34,35,36].

Drug	Duration Class	Route of Administration	Half-Life (t_1_/_2_)	Volume of Distribution	Metabolism	Route of Excretion	Approved Dose –T2DM or Obesity	FDA-Approved Indications	Structural Characteristics
Exenatide	Short-acting	s.c.	~2.4 h	Vd ≈ 28 L	Proteolytic degradation	Renal	5–10 µg twice daily	T2DM	C_149_H_234_N_40_O_47_S
Lixisenatide	Short-acting	s.c.	~2–4 h	Vd ≈ 100 L	Proteolytic degradation	Renal	10 µg then 20 µg once daily	T2DM	C_215_H_347_N_61_O_65_S
Exenatide (ER)	Long-acting	s.c. (depot)	~2 weeks	Vd ≈ 24 L	Proteolytic degradation	Renal	2 mg once weekly	T2DM	C_184_H_282_N_50_O_60_S
Liraglutide	Long-acting	s.c.	~13 h	Vd ≈ 11–17 L	Proteolytic degradation	Renal/biliary	up to 1.8 mg daily -obesity—up to 3.0 mg daily	T2DM, obesity (2014)	C_172_H_265_N_43_O_51_
Dulaglutide	Long-acting	s.c.	~5 days	Vd ≈ 17–19 L	Proteolytic degradation	Renal	0.75–1.5 mg once weekly	T2DM	C_2646_H_4044_N_704_O_836_S_18_
Semaglutide (s.c.)	Long-acting	s.c.	~7 days	Vd ≈ 12.5 L	Proteolytic degradation	Renal/biliary	0.25 mg then 0.5 mg once weekly -obesity—2.4 mg once weekly	T2DM, obesity (2021)	C_187_H_291_N_45_O_59_
Semaglutide (oral)	Long-acting	oral	~24 h	Vd ≈ 8 L	Proteolytic degradation	Renal/biliary	3 mg then 7 mg once daily	T2DM	C_187_H_291_N_45_O_59_
Albiglutide	Long-acting	s.c.	~6–8 h	Vd ≈ 11 L	Proteolytic degradation	Renal	30–50 mg once weekly	T2DM	C_148_H_224_N_40_O_45_

Abbreviations: s.c.—subcutaneous; Vd—volume of distribution; s.c.: subcutaneous administration (injection under the skin); Vd: volume of distribution; a pharmacokinetic parameter representing the apparent theoretical volume into which a drug is distributed to achieve the observed plasma concentration.

**Table 2:** WHO criteria for diagnosing overweight and obesity for adults, children, and adolescents [52].

Age Group	Diagnostic Criterion	Overweight	Obesity
Adults (≥18 years old)	Body mass index (BMI = body weight [kg]/height^2^ [m^2^]	BMI ≥ 25 kg/m^2^	BMI ≥ 30 kg/m^2^
Children (<5 years old)	Weight-to-height ratio relative to the median WHO Child Growth Standards	>+2 SD from the median	>+3 SD from the median
Children and adolescents (5–19 years old)	BMI for age and gender relative to the WHO Growth Reference Median	>+1 SD from the median	>+2 SD from the median

**Table 3:** Studies confirming the role of GLP-1 receptor agonists (GLP-1RA) across different systems, organized by therapeutic area.

Year	Type of Study	Study Group	Treatment Used	Duration of the Study	Main Conclusion	Field of Medicine	
2022	Meta-analysis of RCTs	67,769 with T2D; 34,536 received GLP-1 RAs	GLP-1 receptor agonists vs. placebo	Median follow-up varied by trial 16–65 months)	-GLP-1 RAs reduce risk of MACE, cardiovascular death, and all-cause mortality	Cardiology/Endocrinology	[67]
2024	Meta-analysis	Adults with T2D in RCTs (39,246 participants across 76 trials)	15 different GLP-1 receptor agonists compared with placebo or each other	≥12 weeks per trial (min follow-up)	-All GLP-1 RAs improved glycemic control and weight; tirzepatide was best for HbA_1_c and fasting glucose; CagriSema was best for weight loss; safety concerns re: GI adverse events at high doses	Endocrinology/Diabetes	[42]
2025	Phase 2/3 RCT	559 adults with early T2D	Orforglipron (oral small-molecule GLP-1 receptor agonist) administered once daily in three doses: 3 mg, 12 mg, 36 mg, randomized in a 1:1:1:1 ratio vs. placebo	40 weeks	-Orforglipron significantly improved glycemic control and reduced body weight vs. placebo; generally well tolerated	Endocrinology/Diabetes	[49]
2021	Phase 2b RCT	Adults with overweight/obesity and T2D (834 participants): cotadutide 100 µg (100), 200 µg (256), 300 µg (256), liraglutide 1.8 mg (110), placebo (110)	Cotadutide once daily subcutaneous (100 µg, 200 µg, 300 µg) vs. placebo; open-label liraglutide 1.8 mg	54 weeks	-Cotadutide significantly improved HbA_1_c and reduced body weight vs. placebo; the 300 µg dose also improved hepatic metabolism and weight loss vs. liraglutide	Endocrinology/Diabetes	[50]
2021	Phase 3 RCT	Adults with overweight or obesity (*n* = 1961): Semaglutide group ≈ 1306, Placebo group ≈ 655	Once-weekly subcutaneous semaglutide 2.4 mg + lifestyle intervention vs. placebo + lifestyle	68 weeks	Semaglutide 2.4 mg significantly reduced body weight vs. placebo; more participants achieved ≥5%, ≥10%, ≥15% weight loss and improved cardiometabolic markers	Obesity/Endocrinology	[61]
2023	Phase 2 RCT	272 adults with obesity (BMI ≥ 30) or overweight + ≥1 weight-related condition, without diabetes	Oral orforglipron once daily at 12 mg, 24 mg, 36 mg, or 45 mg vs. placebo	36 weeks	Orforglipron significantly reduced body weight vs. placebo, with 46–75% ≥10% loss vs. 9% placebo; improved cardiometabolic measures; GI side effects common	Obesity/Endocrinology	[62]
2023	Phase 2 RCT	Adults (*n* = 338) with BMI ≥ 30 kg/m^2^, or BMI 27 to <30 kg/m^2^ with at least one weight-related comorbidity (without T2D).	Weekly subcutaneous retatrutide (1, 4, 8, or 12 mg, with initial titration) vs. placebo	48 weeks	Retatrutide produced substantial dose-dependent weight loss vs. placebo.	Obesity/Endocrinology	[65]
2016	Clinical RCT	3297 adults with T2D, age > 50	Semaglutide 0.5 mg or 1 mg s.c. (*n* = 1648)/week vs. placebo (*n* = 1649)	2 years (104 weeks)	-26% reduction in the risk of MACE in patients with high cardiovascular risk T2D	Cardiovascular	[73]
2022	Meta-analysis of RCTs	18 RCTs, 1580 adults patients with type 2 diabetic nephropathy (T2DN)	Liraglutide at a dose of 0.6 to 1.8 mg/day (*n* = 786) vs. placebo (*n* = 794)	from 4 to 24 weeks	-Decrease in: UACR, serum creatinine (Scr), cystatin C (CysC); glucose (including FBG, PBG, HbA1c), BMI, inflammatory markers (TNF-α, IL-6); -No significant change in BUN/eGFR	Nephropathy	[77]
2023	Preclinical	Mice with diabetic nephropathy	Liraglutide 400 µg/kg/day, s.c. (*n* = 8) vs. insulin degludec (*n* = 8)	14 weeks	-Increased podocyte survival, decreased: podocyte apoptosis, inflammation, oxidative stress, albuminuria	Nephropathy	[78]
2024	Meta-analysis of RCTs	17 RCTs, 34,419 adults patients, overweight or obese (BMI > 25 kg/m^2^)	Semaglutide 2.4 mg s.c. weekly, liraglutide 3 mg s.c. daily, oral semaglutide, oral orforglipron (1 RCT); tirzepatide s.c. weekly, beinaglutide 0.2 mg s.c. (*n* = 19,983) vs. placebo (*n* = 14,436)	from 16 to 160 weeks	-Decrease in: Any cardiovascular events, body weight, improvement in metabolic risk markers	Cardiovascular	[79]
2023	Clinical RCT	17,604 patients, median age 61.6	Semaglutide 2.4 mg s.c. weekly (*n* = 8803) vs. placebo (*n* = 8801)	5 years	-Decrease: MACE by 20%, body weight	Cardiovascular	[80]
2024	Meta-analysis of RCTs	24 RCTs, 94,547 patients, median age 58.22	GLP-1 agonists: dulaglutide, efpeglenatide, albiglutide, lixisenatide, exenatide, semaglutide, and liraglutide (oral or subcutaneous once daily or weekly) (*n* = 50,033) vs. placebo (*n* = 44,514)	from 1 to 5.4 years	-Reduction in the risk of MACE -Reduction in cardiovascular deaths -Reduction in heart attacks and strokes -Lower risk of hospitalization due to heart failure.	Cardiovascular	[82]
2019	Preclinical	Human RA fibroblast-like synoviocytes	Lixisenatide (at a concentration of 10 and 20 nM)	24–48 h (cell culture)	-Reduction in: proinflammatory cytokines (TNF-α, IL-6, IL-8), MMP activity, -Inhibition of NF-κB/JNK/AP-1 pathways, -Mitochondrial protection	Immunomodulatory	[90]
2025	Clinical RCT	31 adult patients	Semaglutide 1.0 mg weekly + metformin (*n* = 15) vs. control only metformin (*n* = 16)	12 weeks	-Reduction in the severity of psoriatic lesions (lower PASI) (lower DLQI) -Improvement in inflammation (decrease in CRP, IL-6) Weight loss -Improvement in metabolic profile (LDL, glycemia)	Immunomodulatory	[93]
2018	Preclinical	Rats with 6-hydroxydopamine-induced dopaminergic neurodegeneration	PT302 (long-acting Exendin-4) s.c.	47 days	-Reduced degeneration of dopaminergic neurons -Improved motor function -Neuroprotective effect	CNS-related	[105]
2024	Clinical RCT	156 patients, aged 40 to 75 Parkinson’s disease diagnosed within 3 years	Lixisenatide 10 or 20 µg/day s.c. (*n* = 78) vs. placebo (*n* = 78)	12 months	-Decrease progression of motor disability (MDS-UPDRS III)	CNS-related	[106]
2017	Clinical RCT	62 patients, aged 25 to 75 and Parkinson’s disease.	Exenatide 2.0 mg/s.c. weekly (*n* = 32) vs. placebo (*n* = 30)	60 weeks	-Slower motor progression (MDS-UPDRS)	CNS-related	[107]
2025	Clinical RCT	194 patients, aged 25–80 years, with Parkinson’s disease.	Exenatide 2.0 mg/s.c. weekly (*n* = 97) vs. placebo (*n* = 97)	96 weeks	-Potential disease-modifying effect, slower motor decline	CNS-related	[108]
2023	Meta-analysis	Animal and cell models of Alzheimer’s disease	GLP-1RA	-	-Reducing Aβ deposition and tau protein hyperphosphorylation in the brains of animals with Alzheimer’s disease -Neuroprotective effect -Inflammation modulation	CNS-related	[110]
2016	Clinical RCT	38 patients with mild AD, Age >50 years and <80 years	Liraglutide 0.6–1.8 mg s.c. daily (*n* = 18) vs. placebo (*n* = 20)	26 weeks	-No decline in cerebral glucose metabolism that correlates with disease progression	CNS-related	[111]
2024	Real-world data (RWD)	1,094,761 new patients using antidiabetic drugs	Semaglutide (*n* = 17,104) vs other antidiabetic drugs (including other GLP-1RAs) (*n* = 1,077,657)	3 years	-Reducing the risk of a first diagnosis of Alzheimer’s disease by 40–70%	CNS-related	[112]
2026	Clinical RCT	206 patients with AD, age > 50 years	Liraglutide 0.6–1.8 mg s.c. daily (*n* = 103) vs. placebo (*n* = 103)	12 months	-No significant difference in brain glucose metabolism -Slower cognitive decline and less brain volume loss.	CNS-related	[113]
2025	Retrospective cohort study	14,092 adult patients with multiple sclerosis	GLP1-RA s.c. and p.o. (*n* = 7046) vs. placebo (*n* = 7046)	-	-Reduced risk of MS progression	CNS-related	[116]
2025	Retrospective cohort study	49 patients with multiple sclerosis taking GLP-1, mean age 54 years	GLP-1 RA	about 15.6 months	-GLP-1 agonists are safe in MS patients -Significant weight loss	CNS-related	[117]
2015	Pilot clinical study	44 obese patients, mean age 34 ± 9 years,	Liraglutyd 0.6–3 mg s.c. vs. placebo	12 weeks	-Significant improvement in binge eating, -Reduction in body weight, BMI, waist circumference, systolic blood pressure, fasting glucose, and total cholesterol	CNS-related	[126]
2025	Real-world evidence (RWE)	86,632 patients > 18 years of age with obesity or overweight, without a history of cancer	GLP-1RA exposure (different drugs in the class, e.g., semaglutide, liraglutide) (*n* = 43,317) vs. placebo (*n* = 43,315)	-	-Reduced overall cancer risk, especially endometrial, ovarian, and meningioma cancer, in patients who are obese or overweight	Oncology	[127]
2024	Real-world evidence (RWE)	1,221,218 new patients using antidiabetic drugs	GLP-1RA vs. other antidiabetic drugs	15 years	-Reduced risk of CRC in treatment-naive patients with type 2 diabetes, with or without obesity/overweight. -The effect of reduced CRC risk was more pronounced in patients with obesity/overweight.	Oncology	[128]
2025	Real-world evidence (RWE)	1,212,894 patients with T2D	GLP-1RA vs. other antidiabetic drugs	-	-Reduced risk of gastrointestinal cancers in people with type 2 diabetes	Oncology	[129]
2023	Real-world evidence (RWE)	2562 patients with thyroid cancer who were compared with 45,184 without diagnosed thyroid cancer	GLP-1RA vs. other antidiabetic drugs	-	-Increased risk of developing thyroid cancer and medullary thyroid cancer, especially in patients after 1–3 years of treatment.	Oncology	[130]
2024	Real-world data	1,651,452 patients with T2D, new patients using antidiabetic drugs, mean age 59.8	GLP-1RA (*n* = 149,690) vs. other antidiabetic drugs (insulin, metformin) (*n* = 1,527,652)	15 years	-T2D patients treated with GLP-1RAs compared with insulin had a significant risk reduction among 10 of 13 cancers studied, including esophageal, colon, endometrial, gallbladder, kidney, liver, ovarian, and pancreatic cancers, meningioma and multiple myeloma.	Oncology	[133]
2025	Preclinical	10 samples of oral squamous cell carcinoma (OSCC) and 10 healthy oral epithelial tissues + mouse model	Addition of semaglutide to OSCC tissues and NOK (normal oral keratinocyte) tissues	<21 days	-GLP-1R receptor expression is significantly elevated in oral squamous cell carcinoma (OSCC) cells compared to normal oral keratinocyte (NOK) cells. -Semaglutide inhibited OSCC cell proliferation, migration, and invasion and induced apoptosis (activation of the P38 MAPK signaling pathway).	Oncology	[134]
2024	Meta-Analysis of RCTs	64 RCTs, 84,627 patients with obesity or T2D, mean age 56 years	GLP-1RA (*n* = 46,228) vs. placebo/other antidiabetic drugs (*n* = 38,399)	>52 weeks	-Increased risk of overall thyroid cancer	Oncology	[137]
2025	Retrospective cohort study	351,913 patients, mean age 65.3 years	GLP-1RA (*n* = 41,112) vs. other antidiabetic drugs (DPP4i, SGLT2i, sulfonylurea) (*n* = 310,801)	-	-Low absolute risk of thyroid cancer -Small increase in relative risk limited to the first year of therapy, likely related to increased frequency of ultrasound examinations rather than actual oncological risk	Oncology	[139]

## Data Availability

No new data were created or analyzed in this study. Data sharing is not applicable to this article.
